# The *cnf1* gene is associated with an expanding *Escherichia coli* ST131 *H*30Rx/C2 subclade and confers a competitive advantage for gut colonization

**DOI:** 10.1080/19490976.2022.2121577

**Published:** 2022-09-25

**Authors:** Landry L. Tsoumtsa Meda, Luce Landraud, Serena Petracchini, Stéphane Descorps-Declere, Emeline Perthame, Marie-Anne Nahori, Laura Ramirez Finn, Molly A. Ingersoll, Rafael Patiño-Navarrete, Philippe Glaser, Richard Bonnet, Olivier Dussurget, Erick Denamur, Amel Mettouchi, Emmanuel Lemichez

**Affiliations:** a Institut Pasteur, Université Paris Cité, CNRS UMR6047, INSERM U1306, Unité des Toxines Bactériennes, Département de Microbiologie, Paris, France; b Université Paris Cité et Université Sorbonne Paris Nord, INSERM U1137, IAME, Paris, France; c Laboratoire Microbiologie-hygiène, AP-HP, Hôpital Louis Mourier, Colombes, France; d Institut Pasteur, Université Paris Cité, Bioinformatics and Biostatistics Hub, Paris, France; e Institut Pasteur, Department of Immunology, Mucosal Inflammation and Immunity group, Paris, France; f Université Paris Cité, Institut Cochin, CNRS UMR8104, INSERM U1016, Paris, France; g Institut Pasteur, Université Paris Cité, CNRS UMR6047, Unité Ecologie et Evolution de la Résistance aux Antibiotiques, Département de Microbiologie, Paris, France; h UMR INSERM U1071, INRA USC-2018, Université Clermont Auvergne, Clermont-Ferrand, France; i Centre National de Référence de la Résistance aux Antibiotiques, Centre Hospitalier Universitaire, Clermont-Ferrand, France; j Institut Pasteur, Université Paris Cité, CNRS UMR6047, Unité de Recherche Yersinia, Département de Microbiologie, Paris, France; k AP-HP, Laboratoire de Génétique Moléculaire, Hôpital Bichat, Paris, France

**Keywords:** Escherichia coli, ExPEC, ST131, CNF1, rho GTPases, gastrointestinal tract, colonization, UTI

## Abstract

Epidemiological projections point to acquisition of ever-expanding multidrug resistance (MDR) by *Escherichia coli*, a commensal of the digestive tract and a source of urinary tract pathogens. Bioinformatics analyses of a large collection of *E. coli* genomes from EnteroBase, enriched in clinical isolates of worldwide origins, suggest the Cytotoxic Necrotizing Factor 1 (CNF1)-toxin encoding gene, *cnf1*, is preferentially distributed in four common sequence types (ST) encompassing the pandemic *E. coli* MDR lineage ST131. This lineage is responsible for a majority of extraintestinal infections that escape first-line antibiotic treatment, with known enhanced capacities to colonize the gastrointestinal tract. Statistical projections based on this dataset point to a global expansion of *cnf1*-positive multidrug-resistant ST131 strains from subclade *H*30Rx/C2, accounting for a rising prevalence of *cnf1*-positive strains in ST131. Despite the absence of phylogeographical signals, *cnf1*-positive isolates segregated into clusters in the ST131-*H*30Rx/C2 phylogeny, sharing a similar profile of virulence factors and the same *cnf1* allele. The suggested dominant expansion of *cnf1*-positive strains in ST131-*H*30Rx/C2 led us to uncover the competitive advantage conferred by *cnf1* for gut colonization to the clinical strain EC131GY ST131-*H*30Rx/C2 versus *cnf1*-deleted isogenic strain. Complementation experiments showed that colon tissue invasion was compromised in the absence of deamidase activity on Rho GTPases by CNF1. Hence, gut colonization factor function of *cnf1* was confirmed for another clinical strain ST131-*H*30Rx/C2. In addition, functional analysis of the *cnf1*-positive clinical strain EC131GY ST131-*H*30Rx/C2 and a *cnf1*-deleted isogenic strain showed no detectable impact of the CNF1 gene on bacterial fitness and inflammation during the acute phase of bladder monoinfection. Together these data argue for an absence of role of CNF1 in virulence during UTI, while enhancing gut colonization capacities of ST131-*H*30Rx/C2 and suggested expansion of *cnf1*-positive MDR isolates in subclade ST131-*H*30Rx/C2.

## Introduction

Extraintestinal pathogenic *Escherichia coli* (ExPEC) form a heterogenic phylogenetic group characterized by the presence of specific virulence factors (VFs) conferring elevated risks of contracting severe forms of extra-intestinal infections, such as urinary tract infections (UTI). UTI are common infections that affect more than 150 million individuals, annually, and are the second cause of antibiotic prescription.^1^ Clinical studies document a high prevalence of the cytotoxic necrotizing factor 1 (*cnf1*)-encoding gene in uropathogenic strains of *E. coli* (UPEC), which belong to the larger group of ExPEC, and its presence in the microbiota of healthy patients.^2–4^ CNF1 is a paradigm of bacterial deamidase AB toxins activating Rho GTPases.^5–8^ The *cnf1* gene belongs to the prototypic pathogenicity island (PAI) II_J96_ from the O4:K6 *E. coli* strain J96, that also contains an alpha-hemolysin (HlyA) encoding operon, a UclD adhesin tipped F17-like chaperone-usher (CU) fimbriae, and the PapGII adhesin tipped pyelonephritis-associated pili (pap) operon.^9,10^ Despite hypotheses that CNF1 plays a role in urovirulence,^3^ attempts to define fitness advantages conferred by this toxin in mouse models of UTI have led to opposing conclusions.^11–14^ UTI are inflammatory diseases, although whether CNF1 modulates inflammation, including neutrophil infiltration, into the bladder warrants clarification.^11–13^ This is particularly of interest as, in an animal model of bacteremia, CNF1 exerts a paradoxical host-protective effect antagonized by the action of the genetically associated alpha-hemolysin, further blurring the role of CNF1 in pathogenesis.^15–17^ Cell biology studies established that CNF1 confers high invasive capacities of epithelial cells to *E. coli*, similar to other Rho GTPase activating factors found in *Enterobacteriaceae*.^18,19^ Three types of CNF-like toxins have been described in *E. coli* strains, sharing high amino acid sequence identities.^20–23^ However, isolates expressing the CNF2 and CNF3 toxins are rarely detected in extraintestinal infections in humans. In the clinic, CNF1 is not linked to specific pathophysiological outcomes, in contrast to other known bacterial AB toxins from *E. coli*, such as Shiga-like toxins or the heat-labile toxin.

*E. coli* represents the predominant facultative aerobic bacteria of the gut microbiota, as well as an extraintestinal opportunistic pathogen.^24,25^ The gut is a known reservoir for uropathogenic bacteria, including, notably extended-spectrum beta-latamase (ESBL)-producing *E. coli*.^26–29^ Only a few sequence types (STs) within the *E. coli* population account for more than half of all *E. coli* strains responsible for extraintestinal infections not causally related to antibiotic resistance.^24,30^ The globally disseminated *E. coli* ST131 has emerged as the predominant lineage responsible for worldwide dissemination of the ESBL encoding gene *bla*_CTX-M-15_ and the rise of multidrug resistant MDR extraintestinal infections.^31,32^ This well-defined sequence type is structured into three different clades, with the fluoroquinolone-resistant clade C strains subdivided into two subclades comprised of *H*30R/C1 and the dominant expanding *H*30Rx/C2, frequently carrying *bla*_CTX-M-15_.^33–35^

One reason for the unprecedented success of *E. coli* ST131-*H*30 clade C may be its intrinsic capacity to persist in the gastrointestinal tract (GIT) in competition with other strains of *E. coli*.^27,36–38^ Enhanced colonization capacities of the GIT by *E. coli* ST131 may promote inter-individual transmission, favoring its dissemination in the human population and other hosts, as compared to other lineages.,^27,39–41^ as well as account for a lack of a phylogeographical signal among these strains.^42^ The remarkable fitness of this lineage strongly supports the idea of a step-wise acquisition of factors promoting GIT colonization, potentially scattered among UPEC populations, as well as promoting bacterial virulence or pathogenicity in the context of extraintestinal infections.^43^

To better appreciate *cnf1* dynamics, we performed a large-scale screening of the toxin gene distribution in a large dataset of *E. coli* genomes deposited in EnteroBase.^44^ The observed increase of *cnf1*-positive strains in the ST131-*H*30Rx/C2 lineage led us to hypothesize that *cnf1* may confer a competitive advantage to colonize the GIT. Indeed, the wildtype strain EC131GY from lineage ST131-*H*30Rx/C2 outcompeted a *cnf1*-deleted variant when concurrently inoculated into the GIT, arguing for a role of CNF1 in EC131GY selection within the gut that might be linked to CNF1 deamidase activity on Rho GTPases to promote tissue invasion. Surprisingly, we observed no differences in fitness or inflammation in monoinfections of the urinary tract linked to the presence or absence of *cnf1*. Collectively, these data support although CNF1 does not impact host response to UTI, it acts as an intestinal colonization factor during competition in the GIT.

## Results

### Analysis of the distribution of cnf genes in a large collection of E. coli genomes

At the start of this study, we mined large genomic datasets from EnteroBase to gain more insight into the distribution of the *cnf1* gene and its close homologs in the *E. coli* population.^44^ EnteroBase represents an integrated software environment widely used to define the population structure of several bacterial genera, including pathogens. Quantitative information on the collection of 141,234 *E. coli* genomes deposited in EnteroBase are reported in supplementary Figure S1. This collection, starting in 1900, aggregates genomes from strains collected worldwide, but mainly from Europe and North America, and from a wide range of sources but primarily human clinical isolates (Sup. Figure S1a, S1b, S1c). Using a Hidden Markov Model (HMM) approach coupled to amino acid pairwise distance calculation, we retrieved *cnf*-like positive strains and characterized each type of *cnf* sequence. In total, we identified 6,411 *cnf*-positive strains (4.5% of all *E. coli* isolates) with a remarkable dominance of *cnf1* (87.8%, *n* = 5,634), as compared to *cnf2* (8.6%, *n* = 554) and *cnf3* (3.5%, *n* = 223). These strains displayed only one type of *cnf-*like encoding gene. The prevalent *cnf1* gene in this genomic dataset was widely distributed among isolates of all origins but most notably in the groups denoted humans (5.4% of *n* = 48,518 human isolates) and companion animals (24.1% of *n* = 2,652 companion animal isolates) (Sup. Figure S1c).

**Figure 1.**
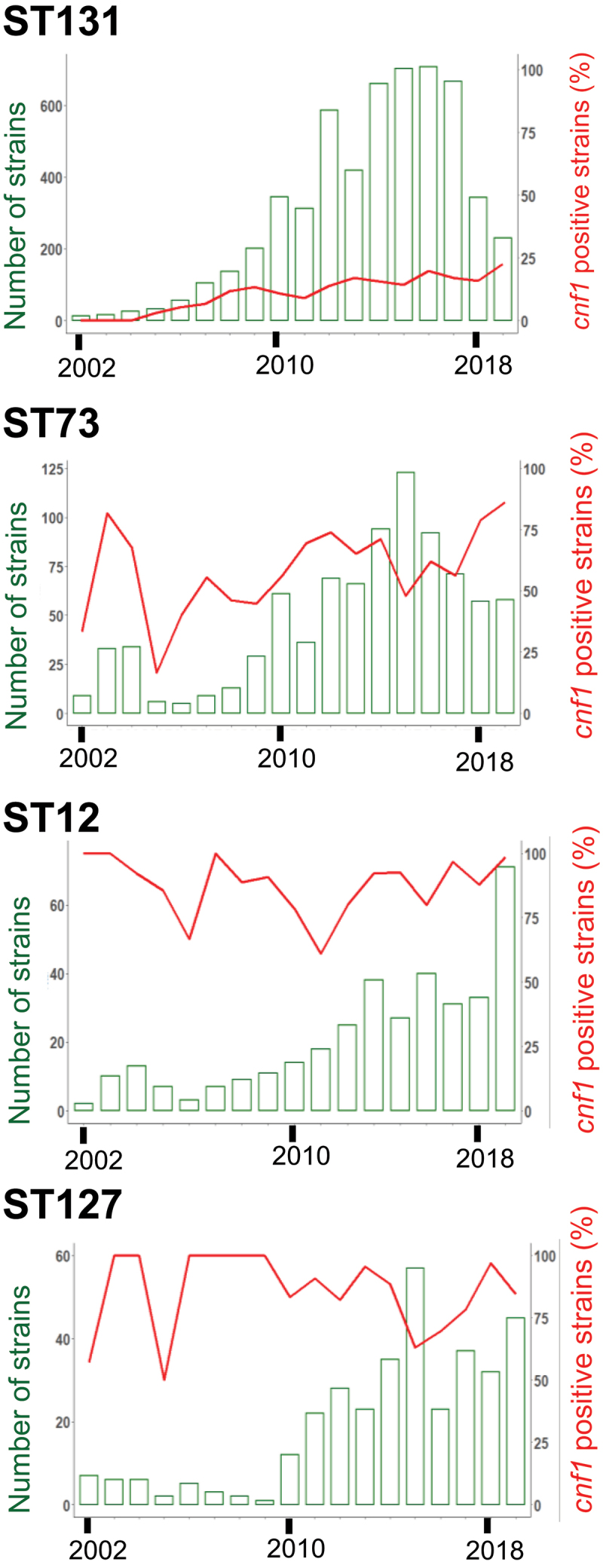
**Prevalence overtime in representative *E. coli* sequence types bearing *cnf1.*** Bar chart show number of *E. coli* strains from ST131, ST73, ST12 and ST127 isolated each year during the period 2002–2019, left y-axis. Percentages of *cnf1*-positive strains per year, right y-axis.

We next studied the distribution of *cnf1* among *E. coli* phylogenetic groups and sequence types (STs). The *cnf1* gene was preferentially associated with isolates from the phylogroup B2, representing 24.3% of *n* = 22,305 retrieved genome sequences (Sup. Figure S1d). We observed a tight association of *cnf1* with the most frequently encountered ExPEC STs (Table 1) (*P* < 2.2 10^−16^, Chi-square association test). Notably, a majority of the 5,634 *cnf1*-positive strains segregated among four STs: ST131 (24.5% of *cnf1*-positive strains, *n* = 1,382), ST73 (23.2%, *n* = 1,308), ST12 (12.4%, *n* = 699), and ST127 (10.7%, *n* = 601). The remaining 29.2% of *cnf1*-positive strains were widely distributed among 266 other STs. Interestingly, we noticed a steady increase of the percentage of *cnf1-*positive strains in the *E. coli* ST131 lineage from 13% in 2009 to 23% in 2019 (Figure 1), while this percentage fluctuated around high values in ST73, ST12, and ST127. This analysis revealed a close association of *cnf1* with common ExPEC lineages and a surprising convergent distribution of *cnf1* in the four lineages ST131, ST73, ST12, and ST127.

**Table 1.** **Distribution of phylogroups and sequence types among *E. coli cnf*-positive strains from EnteroBase**. The total number and the percentage of each phylogroup and most dominant sequence types (STs) among *cnf*-positive strains are indicated.

Phylogroups	ST	Number of strains					Percentage of Phylogroup or Sequence type in CNF-positive strains		
		All	CNF+	CNF1+	CNF2+	CNF3+	CNF1	CNF2	CNF3
A	Total A	34,982	51	0	28	23	0	5.05	10.31
	ST10	8,748	24	0	17	7	0.0	3.1	3.1
	ST342	325	16	0	0	16	0.0	0.0	7.2
B1	Total B1	37,262	527	96	373	58	1.7	67.3	26.0
	ST101	938	93	24	69	0	0.4	12.5	0.0
	ST392	79	66	0	66	0	0.0	11.9	0.0
	ST58	1,487	44	9	35	0	0.2	6.3	0.0
	ST29	496	35	0	0	35	0.0	0.0	15.7
	ST2217	46	31	0	31	0	0.0	5.6	0.0
	ST5738	24	23	0	23	0	0.0	4.2	0.0
	ST21	5,082	10	0	0	10	0.0	0.0	4.5
	ST343	134	2	0	0	2	0.0	0.0	0.9
	ST2836	63	2	0	0	2	0.0	0.0	0.9
	ST4063	3	2	0	0	2	0.0	0.0	0.9
B2	Total B2	22,305	5,478	5,414	63	1	96.1	11.4	0.4
	ST131	9,242	1,383	1,382	0	1	24.5	0.0	0.4
	ST73	2,071	1,308	1,308	0	0	23.2	0.0	0.0
	ST12	809	699	699	0	0	12.4	0.0	0.0
	ST127	709	601	601	0	0	10.7	0.0	0.0
	ST372	366	206	206	0	0	3.7	0.0	0.0
	ST95	1,882	173	147	26	0	2.6	4.7	0.0
	ST141	360	164	164	0	0	2.9	0.0	0.0
	ST998	175	149	149	0	0	2.6	0.0	0.0
	ST80	152	109	105	4	0	1.9	0.7	0.0
	ST537	50	35	35	0	0	0.6	0.0	0.0
	ST647	28	26	0	26	0	0.0	4.7	0.0
C	Total C	3,465	56	45	10	1	0.8	1.8	0.4
D	Total D	9,905	37	20	13	4	0.4	2.3	1.8
E	Total E	16,391	155	7	14	134	0.1	2.5	60.1
	ST11	13,639	113	0	0	113	0.0	0.0	50.7
	ST5592	5	5	0	0	5	0.0	0.0	2.2
	ST11457	4	4	0	0	4	0.0	0.0	1.8
F	Total F	2,957	38	37	0	1	0.7	0.0	0.4
G	Total G	1,862	34	0	34	0	0.0	6.1	0.0
	ST117	1,383	31	0	31	0	0.0	5.6	0.0
Clade I	Total CI	406	18	0	18	0	0.0	3.2	0.0
	ST3057	41	11	0	11	0	0.0	2.0	0.0
Clade II	Total CII	6	0	0	0	0	0.0	0.0	0.0
Clade III	Total CIII	39	0	0	0	0	0.0	0.0	0.0
Clade IV	Total CIV	39	0	0	0	0	0.0	0.0	0.0
Clade V	Total CV	166	0	0	0	0	0.0	0.0	0.0
	Other 358 STs	34,599	1,044	803	215	26	14.3	38.8	11.7

### Cnf1-positive strains segregate into monophyletic groups in ST131 phylogeny

**Figure 2.**
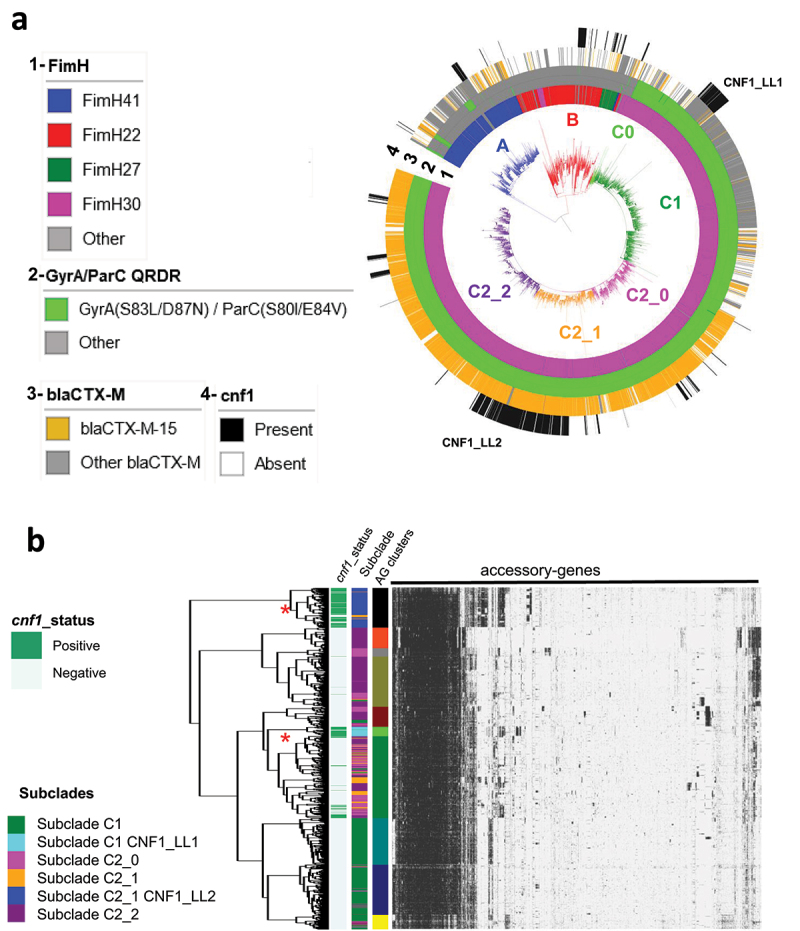
**Dynamic of CNF1-encoding gene in** *E. coli* **ST131 from EnteroBase. A)** Maximum likelihood phylogeny of *E. coli* ST131 from EnteroBase (Sup. Figure S2 for extended information). The phylogeny was constructed with 5,231 genomes for a total of 37,304 non-recombinant core-genome SNPs. The different clades and subclades A, B, C0, C1, C2_0, C2_1, C2_2 are highlighted in blue, red, light green, green, pink, Orange and purple respectively. From inside to outside circles are indicated (1) *fimH* alleles, (2) *gyrA* and *parC* alleles conferring resistance to fluoroquinolones (shown in green), (3) positive strains for *bla*_CTX-M-15_ (shown in Orange) and (4) strains bearing *cnf1* gene (shown in black).) Hierarchical clustering of strains from clade C (*n* = 3981 strains) based on their accessory gene content. The pan-genome is composed of 51,742 genes including 2,672 genes that are present in 98% of the strains. The graph displays the 7,678 genes identified as present in at least 50 and less than 3,930 genomes. The colored annotation indicates (from left to right) the presence of *cnf1* (CNF1_status), subclades (C1, C1 CNF1_LL1, C2_0, C2_1, C2_1 CNF1_LL2, C2_2) and accessory genes cluster (AG_clusters). Large lineages of *cnf1*-positive strains in clades C1 and C2_1 are denoted CNF1_LL1 and CNF1_LL2, respectively. Red stars indicate the two large lineages of *cnf1*-positive strains.

The rising prevalence of *cnf1* in *E. coli* ST131 over time motivated us to study *cnf1* distribution in this ST. EnteroBase contained 9,242 genomes of *E. coli* ST131 at the time of analysis (November 2020). To facilitate genomic analysis, we retained 5,231 genomes isolated from 1967 to 2018. We built a Maximum Likelihood phylogenetic tree based on a total of 37,304 non-recombinant single nucleotide polymorphism (SNPs). Phylogenetic distribution of strains showed an expected dominant population of clade C (76%, *n* = 3,981; 99% *fimH*30), as compared to clade A (11%, *n* = 569; 92% *fimH*41) and B (13%, *n* = 68; 62% *fimH*22) (Figure 2a, Sup. Figure S2a). We also found an expected co-distribution of *parC* (S80I/E84V) and *gyrA* (S83L/D87N) alleles, which confer resistance to fluoroquinolones in most strains from clade C (99.84%, *n* = 3,975 strains), and a tight association of the *bla*_CTX-M-15_ ESBL gene (85%, *n* = 2,194 isolates) with strains from subclade *H*30Rx/C2 (P< 2.2e^−16^, Chi-square association test). The high number of strains gave enough resolution to distinguish two sublineages, C2_1 and C2_2, originating from C2_0 (Figure 2a). From available metadata, we verified the absence of overall geographical and temporal links in the phylogenetic distribution of *E. coli* ST131 strains (Sup. Figure S2b).

We next analyzed the distribution of *cnf1*-positive strains in *E. coli* ST131 phylogeny (*n* = 725, *cnf1*-positive *E. coli*) (Figure 2a, black stripes). The *cnf1*-positive strains were preferentially associated with subclade C2 (*n* = 520) (p < 2.2 10^−16^, Chi-square association test), as compared to subclade C1 (*n* = 101), clade B (*n* = 72), or clade A (*n* = 32) (Figure 2a). Strikingly, most *cnf1*-positive strains segregated into lineages in all clades and subclades with a noticeable distribution of *cnf1*-positive ST131 strains in two large lineages (LL) in *H*30R/C1 (*n* = 101 *cnf1-*positive strains/107 strains in CNF1_LL1) and in *H*30Rx/C2_1 (*n* = 396 *cnf1-*positive strains/425 strains in the CNF1_LL2) (Figure 2a). We then analyzed the diversity of *cnf1* alleles to define their distribution in the phylogeny of ST131 (Sup. Table S1). A similar analysis was performed with the alpha-hemolysin encoding gene, *hlyA*. We found a wide co-distribution of one combination of alleles of *cnf1* (allele P1*_cnf1_*, 85,1%) and *hlyA* (allele P1*_hlyA_*, 77,2%) in *E. coli* ST131 clade A and C, whereas strains from clade B displayed a large range of combinations of various alleles (Sup. Figure S2a). Together, our data point to a clonal expansion of worldwide disseminated ST131-*H*30 strains with the same allele of *cnf1*. This prompted us to perform a clustering analysis of ST131-*H*30 strains according to their accessory gene contents. We generated a pan-genome matrix of 51,742 coding sequences from the *n* = 3,981 strains of clade C. The dataset of accessory genes was built from *n* = 7,678 sequences that were present in at least 50 and no more than 3,931 strains. We conducted a hierarchical clustering of strains and retained 10 distinct accessory gene clusters. Strikingly, this revealed a conservation between phylogenetically defined groups CNF1_LL1 and CNF1_LL2 and groups defined by their accessory gene contents (Figure 2b). Indeed, the hierarchical clustering was most evident for CNF1_LL2, showing a differential enrichment determined with Scoary of *n* = 1,434 genes as compared to other strains from clade C (*P* < 0.05, Bonferroni-adjusted correction). Together, these data point toward intensive group-specific diversification of accessory gene content in *cnf1*-positive clusters in ST131-*H*30.

### E. coli ST131 cnf1-positive strains segregate between two clade-specific virulence profiles

**Figure 3.**
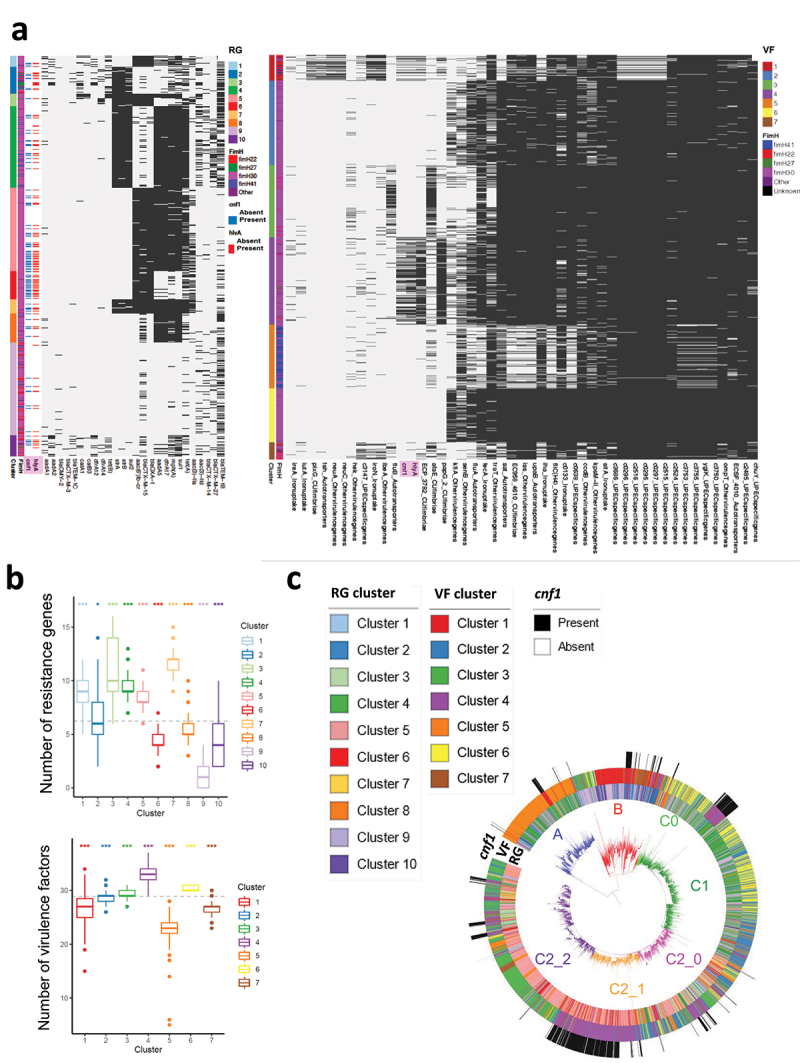
**Co-clustering of acquired antibiotic-resistance gene and virulence factors in *E. coli* ST131. A)** Heatmaps show clusters of antibiotic acquired-resistance gene (RG) (left panel) or virulence gene (VF) (right panel) profiles (Sup. table S2) constructed using a binary latent block model between strains by row and RGs or VFs by column. Black lines indicate the presence of RG or VF in each strain. Annotations are displayed on the right of each heatmap: information about strain clusters and *fimH* alleles together with *hlyA* and *cnf1* carriage. **B)** Box-and-whisker plot showing the distribution of strains according to their content of acquired antibiotic-resistance genes (upper panel) or content of virulence factors (lower panel). The dotted line shows the mean number of RG or VF. All one-versus-all comparisons of VF and RG contents between clusters (*P < 0.05, ***P < 0.001). **C)** RG, VF clusters and *cnf1* carriage are displayed on the *E. coli* ST131 phylogenetic tree. The different clades and subclades A, B, C0, C1, C2_0, C2_1, C2_2 are highlighted in blue, red, light green, green, pink, Orange and purple respectively.

We then defined strain contents in virulence factors (VF) and acquired antibiotic-resistance genes (RG) to perform an unbiased analysis of their distribution into clusters, using a latent block model approach, as described in the materials and methods. The unsupervised clustering procedure identified a total of 10 RG-clusters and 7 VF-clusters (Figure 3a). Differences in number of VFs and RGs among clusters were all significant (Figure 3b). We found that *cnf1*-positive strains were scattered among several RG clusters (Figure 3a, left panel). By contrast, most *cnf1*-positive strains segregated into the VF4 cluster (84% of *cnf1*-positive strains, *n* = 609) with the remaining 16% strains distributed between VF1 (15%) and other VF clusters (1%) (Figure 3a, right panel). In contrast to the scattered distribution of RG-clusters into the phylogeny, we observed a distribution of well-defined groups of VF-clusters (Figure 3c). A majority of *cnf1*-positive strains from clade A and B were part of cluster VF1, whereas *cnf1*-positive strains from clade C were part of cluster VF4. With a median of 33 virulence factors, VF4-positive strains displayed the largest number of virulence factors. The VF1 profile was more specifically defined by the presence of genes encoding the IbeA invasin and IroN Salmochelin siderophore receptor (Sup. Figure S3a). By contrast, the VF4 profile was more specifically defined by *cnf1* and *hlyA* (respectively 54% and 61% in VF4) and also encompassed genes encoding the UclD adhesin that caps the F17-like chaperone-usher (CU) fimbriae cluster and the PapG II adhesin from the pyelonephritis-associated pili (pap) operon (Sup. Figure S3a). ^9,45^ Analysis of several complete sequences of *cnf1*-bearing PAI from ST131-*H*30 showed a conservation of a module containing genes defining VF4 (Sup. Figure S3b). Indeed, elements best defining VF4 were genetically associated and displayed high synteny with *cnf1*-bearing pathogenicity islands (PAI) II_J96_ from the O4:K6 *E. coli* strain J96.

### Cnf1-positive strains display dominant expansion in ST131-H30Rx/C2

**Figure 4.**
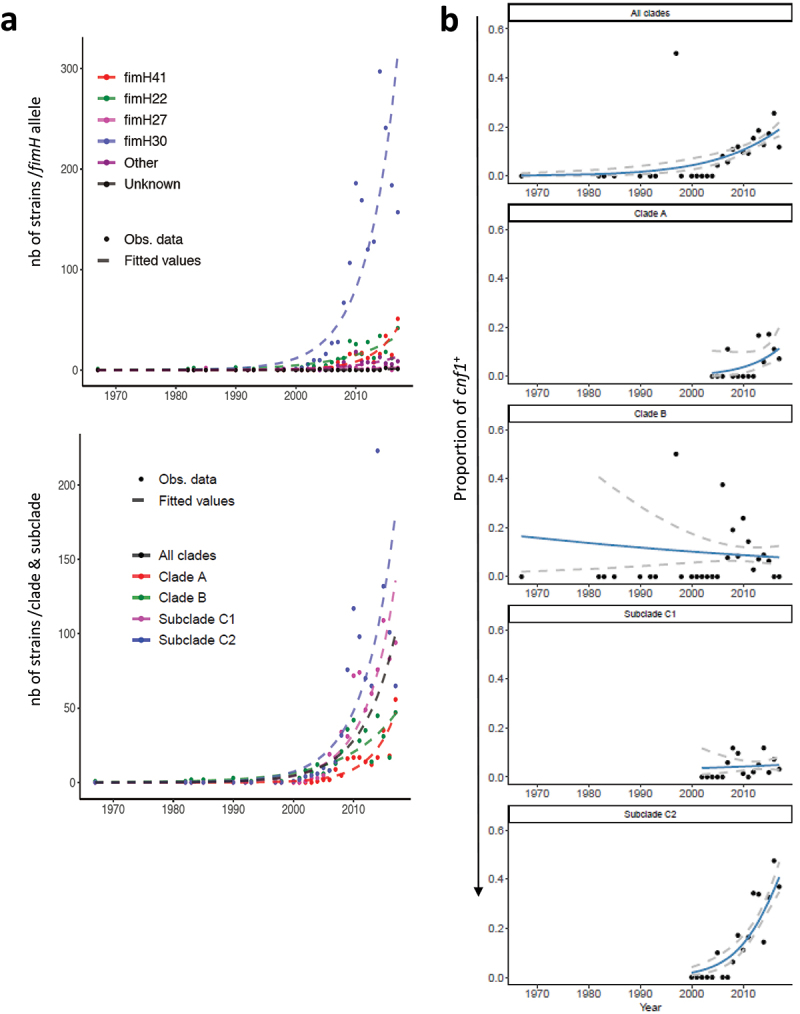
**Increase over the years in the proportion of *cnf1*-positive strains in *E. coli* ST131 *H*30Rx/C2. A)** Distribution of *fimH* alleles (upper panel) or clades/subclades (lower panel) within the study population of *E. coli* ST131. Both figures show observed counts per year (dots) and data fitted lines (dashed lines) with a generalized linear model (Poisson regression). **B)** Increase of the proportion of *cnf1*-positive strains in the whole *E. coli* ST131 population along time (top panel, *P* = 7.41 10^–7^) and by clades and subclades. The black dots represent the observed proportion of *cnf1*-positive strains by year with fitted line of a logistic regression model (blue curves). Dashed gray lines display the 95% confidence intervals. The *P*-values are not significant for clade A (*P* = 0.287), B (*P* = 0.952), *H*30R/C1 (*P* = 0.992) and significant for *H*30Rx/C2 (*P* = 1.25 10^−11^).

We next analyzed the temporal distribution of *cnf1-*positive strains within clades and subclades. Using a Generalized Linear Models (GLM) approach, we first verified within our dataset the increase of *fimH*30-positive isolates over time (clade C) in *E. coli* ST131 that was maximal in *H*30Rx/C2 (*P*  < 2.2 10^−16^, Chi-square association test) (Figure 4a). We also noted a significant increase in the proportion of *cnf1*-positive strains over time in *E. coli* ST131 (Figure 4b, top panel). The GLM was then fitted on years, clades, and subclades. We tested the significance of the year effect and *P*-values were corrected for multiple comparisons using Tukey’s method. The year effect was not significant for clade A, B, or subclade *H*30R/C1 (Figure 4b). Instead, we observed a significant increase of the proportion of *cnf1*-positive strains within *H*30Rx/C2 over time (*P* = 1.25 10^−11^). In addition, the GLM fitted curves predicted that the prevalence of *cnf1*-positive strains within *H*30Rx/C2 subclade would be approximately 50% (confidence interval of 95% [43% to 58%] in 2018; [47% to 64%] in 2019). Predictive values were compared to the prevalence of *cnf1* in ST131 strains isolated in 2018 or 2019 in a second independent dataset up-loaded from EnteroBase in September 2020. The prevalence of *cnf1*-positive strains within the subclade *H*30Rx/C2 was 45% in 2018 and 48% in 2019, confirming the prediction of a dominant expansion of *cnf1*-positive strains within ST131-*H*30Rx/C2.

### Cnf1 confers a competitive advantage for gut colonization in two ST131-H30Rx/C2 strains

**Figure 5.**
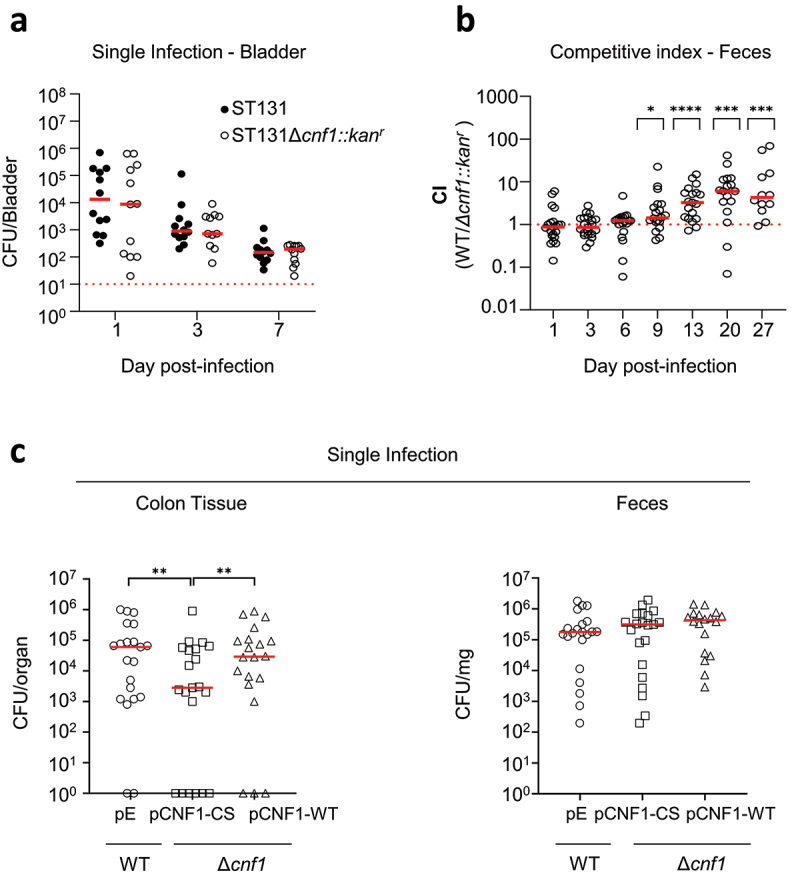
**CNF1 promotes ST131-*H*30Rx/C2 intestinal colonization**. A) For urinary tract infection, mice were infected separately with wildtype EC131GY (WT) and EC131GY Δ*cnf1*::kan^r^ (Δ*cnf1*::kan^r^) via intravesical instillation of the bladder. B-**C**) For GIT colonization, mice were pretreated with streptomycin and subsequently infected concurrently via oral gavage with EC131GY WT and Δ*cnf1*::kan^r^ (B), or with the following stains alone: EC131GY wildtype with an empty vector (WT + pE) as control, EC131GY Δ*cnf1* with a vector encoding catalytically inactive cnf1 (Δ*cnf1* + pCNF1-CS), or EC131GY Δ*cnf1* with a vector encoding cnf1 (Δ*cnf1* + pCNF1-WT) (C). Levels of viable bacteria in bladder homogenates, feces, or colonic tissue after *ex vivo* gentamicin treatment were assessed at indicated times by measuring colony forming units (CFU). Data show CFU per bladder at 1, 3, and 7 d post-infection (**A**), competitive index (CI) in feces at indicated days post-infection (**B**), CFU in colon tissues and feces at 72 hours post-infection (**C**) for each animal and medians (red bar). (**A** and **C**) bladders are *n* = 9–10, two replicates at day 1, 3, 7 and feces CI, *n* = 22–24, three replicates. *P < 0.05, **P < 0.01, ***P < 0.001, ****P < 0.0001 and ns: non-significant by Wilcoxon signed-rank test. (**C**) colon tissues and feces *n* = 20–21, four replicates. A mixed effect model adjusted on the conditions and dates. **P < 0.01 (Δ*cnf1* + pCNF1-CS vs WT + pE), **P < 0.01 (Δ*cnf1* + pCNF1-CS vs Δ*cnf1* + pCNF1-WT), and *P* = 0.98 (WT + pE vs Δ*cnf1* + pCNF1-WT).

The dominant expansion of *cnf1*-positive strains in ST131 *H*30Rx/C2 prompted us to explore whether CNF1 might function as a virulence factor in UTI and a colonization factor in the gastrointestinal tract, a natural environment for *E. coli*. We selected a VF4/*cnf1*-positive strain of *E. coli* ST131 *H*30Rx/C2, here referred to as EC131GY (Sup. Figure S4). This strain displays a prototypic *cnf1*-bearing PAI II_J96_ (Sup. Figure S3b). We generated an EC131GY strain in which *cnf1* was replaced with a kanamycin resistance cassette (EC131GY Δ*cnf1::kan*^r^) and verified the absence of CNF1 expression (Sup. Figure S5a). We then verified, *in vitro*, the absence of fitness cost due to the kanamycin resistance cassette as shown by equal growth of the parental and Δ*cnf1::kan*^r^ EC131GY strains, and the absence of competition between the strains when grown together (Sup. Figure S5b and S5c). Next, in mono-microbial bladder infections, we observed no difference in the number of colony-forming units (CFU) between wildtype EC131GY and the Δ*cnf1::kan*^r^ strain at 1, 3, and 7 d post-infection (Figure 5a). In addition, we observed indistinguishable responses in 20 variables of the innate immune response between the two infections at 24 hours post-infection (Sup Figure S6A-S6E). This included no observed difference after infection with either of the two strains in inflammatory cytokine expression, or in proportions of resident macrophage subsets, dendritic cells, monocytes, neutrophils, NK, or lymphoid cell populations (Sup Figure S6B-S6E).

We then explored the impact of *cnf1* on GIT colonization by competitive infection with wildtype EC131GY and EC131GY Δ*cnf1::kan*^r^, using intra-gastric gavage, to model the natural environment in which several strains of *E. coli* are present. Longitudinal measurements of CFU in the feces showed that *cnf1* conferred an advantage to wildtype EC131GY over the EC131GY Δ*cnf1::kan*^r^ isogenic strain for gut colonization from 9 d after oral gavage, which persisted over 27 d (Figure 5b). We confirmed the competitive advantage for gut colonization conferred by *cnf1* in another clinical isolate, BLSE2018-86 from ST131 *H*30Rx/C2, which had an advantage over the *cnf1* mutant from 6 d post-inoculation (Sup. Figure S4 and S7). We next performed a quantitative approach to assess the impact of CNF1 deamidase activity on the efficiency of colon tissue invasion by EC131GY. The strain EC131GY Δ*cnf1* was transformed with plasmids expressing wildtype CNF1 (Δ*cnf1* + pCNF1-WT) or the catalytically-inactive mutant C866S (Δ*cnf1* + pCNF1-CS), while the wildtype CNF1-producing EC131GY was transformed with an empty vector as a control (WT + pE). These strains showed equal growth kinetics *in vitro* (Sup. Figure S5b). Equivalent quantities of each strain were delivered by oral gavage. At an early time point of 72 hours post-infection, we enumerated CFU in feces and colon tissue after *ex vivo* gentamicin treatment of tissues, a membrane non-permeant aminoglycoside. While we observed no difference in CFU in gentamicin-treated colonic tissues between the two strains expressing wildtype CNF1, we observed a significant lower number of CFU for EC131GY Δ*cnf1* expressing the catalytically inactive C866S form of the CNF1 toxin (Figure 5c). We interpret these data to support that invasion of colon tissue by EC131GY is mediated, at least in part, by CNF1 deamidase activity. Together, these data uncover an advantage conferred by CNF1 for GIT colonization by two clinical strains of ST131-*H*30Rx/C2 subclade.

## Discussion

*E. coli* ST131 has rapidly become a globally dominant lineage of ExPEC responsible for UTI that are resistant to antibiotic treatments, globally. Independently of the acquisition of multidrug-resistance genes, advantages ascribed to the ST131 lineage encompass an increased capacity to colonize the GIT, although molecular determinants enhancing gut colonization remain to be defined.^41,46^ Here, we report that *cnf1* enhances the capacity of ST131 *H*30/Rx/C2 to compete with the *cnf1*-negative isogenic strains for gut colonization. Moreover, CNF1 deamidase activity enhances EC131GY capacity to invade colon tissues. These findings represent a change of paradigm for the CNF1 toxin by providing evidence that CNF1 has no detectable impact on inflammation during the first 7 d of UTI and enhances enhances ST131 *H*30/Rx/C2 competitive advantage of gut colonization. Although, more work is needed to clarify the role of *cnf1* in other aspects of UTI virulence, such as the formation of bladder reservoirs or in recurrent UTI, our data clearly assigned to CNF1 a function of gut colonization factor.

In parallel, statistical analysis of more than five thousand isolates of *E. coli* ST131 from EnteroBase, suggests an intercontinental expansion of *cnf1*-positive *H*30Rx/C2 strains among human clinical isolates from subclade C2 and ST131. This hypothesis is supported by the distribution of a large cluster of VF4/*cnf1*-positive H30Rx/C2 strains onto the phylogeny sharing the same alleles of *cnf1* and *hlyA* toxin genes. Indeed, the expansion of a phylogenetic subcluster of ST131-*H*30Rx/C2 strains bearing *cnf1*-positive PAI within the subclade C2 has recently been highlighted for companion dog isolates.^47,48^ Thus, although the EnteroBase *E. coli* dataset likely includes some bias in the sampling of strains with a high abundance of strains responsible for acute human infections and from specific continents, this database is well-adapted to study toxin gene distribution in human clinical isolates of *E. coli*. Further in support of a possible advantage conferred by the acquisition of *cnf1*, we found a high coverage of strains positive for *cnf1* in the common clinical STs, ST73, ST12, and ST127, but not in ST95.^24^ Collectively, these findings suggest that *cnf1* may enhance the capacity of GIT colonization by some ExPEC, in turn contributing to the dissemination of a few *E. coli* lineages and sublineages, such as ST131-*H*30Rx/C2.

The intestinal tract is a key reservoir for ExPEC strains.^49^ A previous study suggested that *E. coli* ST131 has a high capacity to invade human intestinal epithelial cells and persistently colonize mouse GIT in a type I pili-dependent manner.^46^ Our findings, that *cnf1* gives a competitive advantage for GIT colonization and that CNF1 toxin deamidase activity enhances invasion of colonic tissue, raises the interest of defining the relationships between *cnf1* and type I pili for tissue colonization. This includes both bladder and gut tissue colonization. Indeed, our data showing an absence of difference in inflammation during UTI does not rule out a potential effect of CNF1 in bladder tissue colonization and chronic carriage, leading to recurrent UTI. Indeed, cell biology studies showed that CNF1 promotes epithelial cell invasion, including bladder epithelial cells, by *E. coli* through its capacity to activate host Rho GTPase signaling.^19,23,50,51^ In line with this, CNF1 deamidase exacerbates Rho GTPase signaling, and notably Rac1, to promote type I pili-mediated host cell invasion.^52,53^ Enhanced capacities to invade and colonize intestinal tissues may also involve factors encoded within PAI II_EC131GY_-like from ST131 *H*30Rx/C2. Indeed, *cnf1*-bearing PAIs contain a core set of genes encoding the F17-like pili, the P-fimbriae tipped with PapG class II adhesin, and the HlyA toxin, as well as a gene encoding hemagglutinin *in E. coli* K1 (Hek).^54–56^ The *cnf1*-bearing PAIs also include elements of oxidative stress adaptation, namely the methionine sulfoxide reductase complex MsrPQ encoding genes *yedYZ*, which may work against CNF1-generated oxidative stress.^57,58^

Large-scale phylogenetic reconstruction of ST131 genomes from EnteroBase showed an expected phylogenetic distribution within clades and subclades of genetic traits defining this lineage. We report a stable population of *cnf1*-positive strains in H30R/C1 in EnteroBase, contrasting with the expansion of *cnf1*-positive strains in *H*30Rx/C2. Moreover, we observed a high prevalence of *cnf1*-positive strains in a few STs commonly responsible for extraintestinal infections. It will be of interest to decipher the interplay of *cnf1* in gut colonization by *H*30R/C1, as well as ST73, ST12, and ST127 that display lower acquired resistance gene content as compared to *E. coli* strains from ST131 subclade C2.^24,59^ This should help draw the relationship between strain-specific profiles of antibiotic resistance and the function of *cnf1* in gut colonization linked to bacterial dissemination. This may also help define epistatic relationships between *cnf1* function as a gut colonization factor and strain-specific genetic backgrounds, including regulatory factors of *cnf1*-gene expression, toxin secretion, and strain-dependent adaptation to the gut environment including invasion of specific niches in the intestine.^49,60,61^

## Material and methods

### E. coli genomes dataset

The dataset corresponds to 141,234 *E. coli* genome sequences retrieved from EnteroBase (November 2020) (http://enterobase.warwick.ac.uk) ^44^ Strains’ metadata (collection year, continent, source niche of isolation and sequence type) were also retrieved (Sup. Table S3). Assemblies were downloaded in GenBank format and proteomes generated using annotations provided in GenBank files.

### In silico detection and typing of cnf-like toxin encoding genes

The search for *cnf* genes in *E. coli* genomes was carried out with a domain specific Hidden Markov Models (HMM) profile built with 16 representative sequences of CNF1 catalytic domain (Sup. Table S4) using HMMER (http://hmmer.org/) ^62^ Protein sequences from positive hits were extracted from EnteroBase annotated *E. coli* proteomes and submitted to Clustal Omega for the computation of pairwise distances of the sequences, along with representative sequences of CNF-like toxin (CNF1 (AAA85196.1), CNF2 (WP_012775889.1) and CNF3 (WP_02231387.1)). Distances were used to determine the type of toxin with a threshold value of 0.1. In total 2.7% of HMM-positive sequences with a threshold value above 0.1 against all type of CNF-like toxin or below 0.1 against at least two type of CNF-like toxin were excluded from the analysis.

### ST131 dataset structure and phylogenomic analysis

The database used for phylogenetic and statistical analyses consists of whole-genome sequences of *E. coli* ST131 isolates collected by mining EnteroBase from 1967 to 2018.^44^ Leaning on Find ST(s) tool from EnteroBase, we retained a total of 5,231 genome assemblies and associated metadata, including information of the isolation date, country and source of isolates (Sup. table S5). Phylogeny of ST131 isolates was resolved using core non-recombinant SNPs defined with Parsnp (in total 37,304 SNPs)^63^ and Gubbins v2.3.4.^64^ A maximum-likelihood tree was then estimated with RAxML v8.2.8 applying a general time-reversible substitution-model with a gamma distribution rate across sites and with an ascertainment bias correction^65^ and the resulting tree was edited with the interactive Tree of Life (iTol) v4 program.^66^ Chi-square association test was used to evaluate the significant association of *cnf1* and *bla*_CTX-M-15_ with subclade C2.

### Pan-genome analysis

The pangenome of *E. coli* ST131 was estimated using Roary, a high-speed pan genome pipeline analysis tool.^67^ Roary returns as output, the gene presence/absence matrix. The matrix was curated to retain genes present in at least 50 genomes and less than 3931 genomes (7678 sequences), that constituted our accessory genes pool dataset. Hierarchical clustering analysis was then conducted according to the Ward’s minimum variance-derived method. The Ward’s method is a clustering criterion that aggregates observations into clusters to minimize the within-cluster variance. The method was implemented using the pheatmap package in R (cran.r-project.org/web/packages/pheatmap/index.html). The gene presence/absence file generated by Roary was further analyzed using Scoary with a significant Bonferroni-adjusted *P*-value < 0.05 for genes associated to *cnf1*-positive lineages (Sup. Table S8).^68^

### In silico antimicrobial resistance and virulence-associated markers

GyrA and ParC protein sequences were retrieved from the EnteroBase annotated genomes, and aligned with the mafft L-INS-I approach.^69^ After a visual inspection of the alignment, in-house customized perl scripts (https://github.com/rpatinonavarrete/QRDR) were used to identify the amino acids at the quinolone resistance-determining region (QRDR) (positions 83 and 87, and 80 and 84 in GyrA and ParC, respectively). Search for *cnf1* and *hlyA* alleles in ST131 genomes dataset was carried out by Blastn analysis. Sequences were next aligned with Muscle^70^ and curated to remove incomplete sequences. SNPs were then extracted using SNP-sites.^71^ To determine strain specific VF profiles, annotated VFs from UPEC described in^34^ were translated and pBLASTed against ST131 genomes dataset considering only hits with e-value < 10^–5^ and identical matches > 95% (sup. Table S2).^72^ Acquired antibiotic-resistance genes (RGs) in ST131 genomes were defined with ResFinder.^73^

### Co-clustering method

Statistical analyses were performed using R software version 3.6.0. A total of 20 strains from the collection of 5,231 strains of *E. coli* ST131 were removed from the analysis due to incomplete associated metadata. The clustering of strains with specific virulence or acquired antibiotic-resistance gene profiles was performed with binary latent block model, implemented in the R package blockcluster.^74^ The co-clustering of both virulence or resistance genes and strains was performed with a binary latent block model, implemented in the R package blockcluster.^74^ This package implements an Expectation Maximization algorithm to compute the maximum likelihood estimator of the parameters of the mixture of Bernoulli distributions used for co-clustering. As proposed by the authors,^74^ the number of clusters was estimated by maximizing the integrated complete-data likelihood criterion (ICL) on a bidimensional grid of parameters making this unsupervised classification procedure automatic.

### Generalized linear model

Proportion of *cnf1* along time was modeled using a generalized linear model fitted with binomial distribution and logit link. The model was adjusted on the effect of years and clades with an interaction between these two factors. We used the Tukey’s HSD test which adjusts the *P*-values for multiple comparisons (5 comparisons, one by clade and one for gathered clades). First, to test if the evolution of *cnf1* proportion was either specific to each clade or global, the significance of the interaction term was tested with a likelihood ratio test, which compares the above-mentioned model against the null model, with no interaction. Then, we investigated the possible increase of the proportion of *cnf1* within each clade. The significance of the slope coefficient for each clade was tested by computing contrasts of the above model. *P*-values were adjusted for multiplicity using single-step correction method. The distribution of *fimH* alleles and clades/subclades within the study population of *E. coli* ST131 was analyzed with a similar approach, except that a Poisson regression model was used to model counting data. The hypothesis testing strategy to investigate the significance of the increase of *fimH* alleles and clades/subclades along time is discussed above.

### Construction of bacterial strains

The ST131 strain H1-001-0141-G-Y, here referred to as EC131GY, was originally isolated from a patient suffering from bacteremia (Sup. table S6).^75^ The strain is naturally resistant to ampicillin, to cefotaxime (CMI >256 mg/L) and is susceptible to gentamicin (CMI 0.5 mg/L). A streptomycin-resistant isolate was selected and used to engineer the *cnf1* mutant strain. Deletion of *cnf1* gene from the chromosome of EC131GY was achieved by gene replacement with kanamycin resistance (EC131GY Δ*cnf1::kan^r^*) using the Lambda Red recombination system for gene replacement as previously described.^76^ Briefly, primers for amplification of the kanamycin cassette and the flanking FRT regions in pKD4 have been designed to target the first and the last 81 nucleotides of the *cnf1* gene (Sup. table S7). The resulting PCR product was purified using commercial kits (Macherey Nagel). The strain carrying the temperature-sensitive helper plasmid pKOBEG coding for the Lambda red recombinase system was processed as previously described.^76^ The resulting mutants were tested for the gene replacement by PCR with primers listed in the supplementary table S7, and pKOBEG plasmid loss was verified on LB agar plates with chloramphenicol. The kanamycin cassette in EC131GY Δ*cnf1::kan^r^* was removed with pCP20 expressing flippase, as reported in,^77^ to generate EC131GY Δ*cnf1*. Resulting colonies were verified by PCR with primers listed in the supplementary table S7. The *cnf1* gene including its promoter region was cloned *BamH*I and *Kpn*I in pISN1 bearing chloramphenicol resistance,^78^ a gift from Petra Dersch, here referred to as pCNF1-WT (Sup. table S6). The plasmid encoding the catalytically-inactive mutant C866S (pCNF1-CS) was generated by site-directed mutagenesis using oligonucleotides listed in supplementary table S7. The stain BLSE2018-86 was isolated from a patient suffering from UTI.^47^ All mutants were verified for growth in LB by performing growth curves in a FLUOstar Omega microplate reader. Briefly, starting from a fresh overnight culture, bacteria were diluted 1/100 in 5 mL LB supplemented with streptomycin 200 µg/mL. 200 μL of each culture were placed as 5 replicates in a 96 flat bottom plate (Greiner) and incubated for 12 hours at 37°C with 120 rpm orbital shaking. Absorbance at 600 nm was measured every 10 minutes.

### Western blot

Bacterial pellets were collected in RIPA buffer. The lysates were boiled in 1x Laemmli buffer for 5 minutes at 100°C and resolved on 8% SDS-PAGE, transferred to nitrocellulose membrane (GE Healthcare). The proteins were colored with ponceau S (Biorad) and the membrane was blocked with 5% milk in TBS-T (Euromedex). Membranes were incubated with the primary antibody: CNF1 (Santa Cruz sc52655 clone NG8 1/1000), RNA Polymerase (Biolegend 699907 clone NT73 1/1000) and rabbit serum (1/1000) against the conserved amino acids 914–936 of HlyA, as previously described.^79^ Membranes were washed with TBS-T and incubated with horseradish peroxidase (HRP)-conjugated secondary antibodies for 1 h. Signals were observed using Immobion Western Chemiluminescent HRP Substrate (Merck).

### Mouse colonization model

Local Animal Studies Committee and National Research Council approved all procedures used for the mouse experiments described in the present study (APAFIS#26133-202006221228936 v1, 2016–0010). For intravesical infection: urinary tract infection was induced in female C57BL/6 mice aged 6–7 weeks (Charles River), as previously described.^80,81^ Briefly, a single colony of EC131GY or the *cnf1* mutant was inoculated in 10 mL LB medium with antibiotics and incubated at 37°C under static conditions for 18 h. Mice were infected with a total of 10^7^ CFU of bacteria in 50 μL PBS via a urinary catheter under anesthesia. To calculate CFU, bladders were aseptically removed and homogenized in 1 mL of PBS. Serial dilutions were plated on LB agar plates with antibiotics, as required. For gut colonization, groups of female C57BL/6 mice aged 6–7 weeks (Charles River) were pretreated with a single dose of streptomycin (1 g/kg in 200 µL water) *per os* 1 d prior to gavage, as described in^82^ and infected with the strains derived from EC131GY or BLSE2018-86. Mice were infected *per os* with 2 × 10^9^ CFU of each strain either alone or in 1:1 mix (WT: mutant strains) for the competitive index (CI) in 200 μL PBS. Fecal pellets were collected from every individual mouse at indicated times, weighed and homogenized in 500 μL phosphate-buffed saline (PBS) pH 7.2 by vigorous vortexing. CFUs were determined by plating serial dilutions on selective LB agar plates. Strains were prepared for infection as follows: a single colony of EC131GY or BLSE2018-86 or their derivative was inoculated in 10 mL selective LB medium and incubated at 37°C under static conditions for 24 h. Bacteria were then inoculated in 25 mL fresh selective LB medium at 1:1000 dilution and incubated at 37°C under static conditions for 18–24 h. Bacteria were then washed twice in cold PBS, and concentrated in PBS at approximately 2 × 10^9^ CFU per 200 μL. Inocula titers were verified in parallel for each infection. The value of CI was calculated as: CFU WT output strain/CFU mutant output strain, with the verification in each experiment that CFU WT input strain/CFU mutant input strain was very close to 1. A Wilcoxon signed-rank test was performed to assess the statistical significance of differences in CI over time. Statistical analyses were performed with GraphPad Prism 9. CFU in colon tissues were assessed upon treatment *ex vivo* in gentamicin 100 µg/mL for 2 hours. Washed tissues were homogenized in PBS using IKA T25D Ultra Turrax homogenizer and CFU were determined by plating serial dilutions on selective agar. To assess the statistical significance of colonic tissue invasion, a linear mixed model was applied to the Log_10_ values of CFU. This model was adjusted on conditions EC131GY Δ*cnf1* + pCNF1-CS or pCNF1-WT and EC131GY + empty vector (pE) as fixed effect and on the date of experiment as random effect. Comparison performed using contrasts within this model and *P*-values adjusted using Tukey correction in R software.

### Enzyme-linked immunosorbent assay (ELISA)

IL-6, TNF-α, and CXCL1 were measured in bladder tissue homogenates (also used for CFU measurements) using the R&D Systems DuoSet ELISA kits according to manufacturer’s protocols with no changes, except, due to limited sample volumes, 45 μL of experimental samples were used instead of 100 μL.

### Flow cytometry

Mice were sacrificed at 24 hours post-infection (PI) and the bladders removed. Single-cell homogenates were prepared by incubating minced bladders in 0.34 Units/mL Liberase TM (Roche) diluted in PBS at 37°C for 1 hour, with manual agitation every 15 minutes.^80^ Digested tissue was filtered using a 100 μm filter (Miltenyi), washed, blocked with Fc Block (Rat anti-mouse CD16/CD32, BD Biosciences), and immunostained (Supplementary Table S9). Samples were acquired on a BD Fortessa (BD Biosciences) and analyzed using FlowJo Version 10.7.1 software.

## Supplementary Material

Supplemental Material

## Data Availability

Raw data are available at http://enterobase.warwick.ac.uk and processed data in supplementary tables.
