# Supplementary material for: The cnf1 gene is associated with an expanding Escherichia coli ST131 H30Rx/C2 subclade and confers a competitive advantage for gut colonization
Source: Gut Microbes. 2022 Sep 25;14(1):2121577. doi: 10.1080/19490976.2022.2121577 (PMC9519008; doi:10.1080/19490976.2022.2121577)
Supplement: Supplemental Material [file KGMI_A_2121577_SM6574.zip › 2121577_supplementary New/Tsoumtsa_et_al_sup_legends_v18082022.docx]

**Supplemental Material**

**The *cnf1* gene is associated with an expanding *Escherichia coli* ST131 *H*30Rx/C2 sublineage and confers a competitive advantage for gut colonization**

Landry L. TSOUMTSA MEDA^1*^, Luce LANDRAUD^2,3*^, Serena PETRACCHINI^1,4*^, Stéphane DESCORPS-DECLERE^1,5^, Emeline PERTHAME^5^, Marie-Anne NAHORI^1^, Laura RAMIREZ FINN^6^, Molly A. INGERSOLL^6^, Rafael PATIÑO-NAVARRETE^7^, Philippe GLASER^7^, Richard BONNET^8,9^, Olivier DUSSURGET^10^, Erick DENAMUR^2,11^, Amel METTOUCHI^1#^ and Emmanuel LEMICHEZ^1#^

*^1^ Institut Pasteur, Université Paris Cité, UMR CNRS 6047, Inserm U1306, Unité des Toxines Bactériennes, 75015 Paris, France*

*^2^ Université Paris Cité and Université Sorbonne Paris Nord, Inserm, IAME, F-75018 Paris, France*

*^3^ Laboratoire Microbiologie-hygiène, AP-HP, Hôpital Louis Mourier, 92700 Colombes, France*

*^4^ Université de Paris, 75006, Paris, France*

*^5^ Institut Pasteur, Université de Paris, Bioinformatics and Biostatistics Hub, 75015 Paris, France*

*^6^ Institut Pasteur, Mucosal Inflammation and Immunity group, 75015 Paris, France*

*^7^ EERA Unit "Ecology and Evolution of Antibiotic Resistance », Institut Pasteur, Université Paris Cité, UMR 6047 CNRS, Paris, France*

*^8^ Institut National de la Santé et de la Recherche Médicale (UMR 1071), Institut National de la Recherche Agronomique (USC-2018), Université Clermont Auvergne, Clermont-Ferrand, France*

*^9^ Centre National de Référence de la Résistance aux Antibiotiques, Centre Hospitalier Universitaire, Clermont-Ferrand, France*

*^10^ Institut Pasteur, Université Paris Cité, UMR CNRS 6047, Yersinia Research Unit, 75015 Paris, France*

*^11^ AP-HP, Laboratoire de Génétique Moléculaire, Hôpital Bichat, 75018 Paris, France*

* co-first authors

#: corresponding authors :emmanuel.lemichez@pasteur.fr and amel.mettouchi@pasteur.fr

Unité des toxines bactériennes

Institut Pasteur

25 Rue du Docteur Roux
75724 PARIS CEDEX 15d
Tél : +33140613044

**SUPPLEMENTAL FIGURE LEGENDS**

**Sup. Figure 1: Available metadata associated with genomes of *E. coli* from EnteroBase**

**A-B)** Available metadata of *E. coli* isolates from EnteroBase show their distribution according to the period of isolation (A) and geographic origin (B). Red line shows the percentage of *E. coli* strains that were *cnf*-toxins positive over the years. **C-D)** Tables show the percentage of *cnf1*-positive strains for each origin (C) or each phylogroup (D). Total corresponds to the number of strains with available metadata information.

**Sup. Figure 2: Maximum likelihood phylogenetic tree of *E. coli* ST131 genomes**

**A-B)** Maximum likelihood phylogeny of *E. coli* ST131 from EnteroBase with different clades and subclades A, B, C0, C1, C2_0, C2_1, C2_2 highlighted in blue, red, light green, green, pink, orange and purple, respectively. Phylogeny was constructed with 5,231 genomes for a total of 37,304 non-recombinant core-genome SNPs and visualized with iTol. Colour strips surrounding the phylogram represent, from inside to outside circles: somatic (O) and flagellar (H) antigen combination (1) and alleles of *fimH* (2), *gyrA* (3), *parC* (4), *bla*_CTX-M_ (5), *hlyA* (6) and *cnf1* (7) for each strain. **B)** Geographical origin (1) and period of isolation of each strain (2) of *E. coli* ST131 isolates together with *cnf1*-positive strains from large clusters (3) CNF1_LL1 (green) and CNF1_LL2 strains (blue).

**Sup. Figure 3: *cnf1* together with elements of PAI IIJ96 specify VF4 cluster**

**A)** Left graph shows prevalence of virulence factors from VF4 in *E. coli* ST131 study population (x-axis) and the 1,128 VF4-positive strains (y-axis). Similar analysis for VF1-positive strains (right graph). **B)** Comparison of the genomic organization with PAI II_EC131GY_ from EC131GY and 8 other *E. coli* ST131 strains from EnteroBase (ESC_VA2376AA_AS, ESC_JA0942AA_AS, ESC_VA2412AA_AS, ESC_VA2988AA_AS, ESC_LA1508AA_AS, ESC_VA2411AA_AS, ESC_JA0943AA_AS and ESC_JA0947AA_AS), as well as reference PAI II_J96_ from the strain J96 (GCA_000295775.2). Genomic islands containing *cnf1*-encoding gene were defined with IslandPath-DIMOB of Islandviewer 4. Annotated sequences were aligned and visualized using Easyfig. Red lines between PAIs show > 60% blast homologies. Genes located inside PAIs are displayed as colored arrows together with annotation of large operons. Genes or operon encoding the haemagglutinin from *E. coli* K1 (Hek), fimbrial adhesin PapG, F17-like pili, Cytotoxic Necrotizing Factor-1 (CNF1), alpha-hemolysin (HlyA), yedYZ-encoding methionine sulfoxide reductase (MsrPQ), histidine-kinase two-component system (YedVW), contact-dependent growth inhibition (CdiI), cryptic phage-bearing toxin/antitoxin systems CP4-57 and CP4-44, and lysine decarboxylase (CadCBA) are annotated. Other open reading frames are indicated in blue.

**Sup. Figure 4: Phylogenetic tree revealing the position of EC131GY and BLSE2018-86**

Localization of EC131GY and BLSE2018-86 onto the phylogenic tree of *E. coli* ST131 strains from the subclade C2. Phylogeny was constructed using core-genome non-recombinant SNPs of 920 *E. coli* ST131 strains from subclade C2 including EC131GY and BLSE2018-86 genomes. EC131GY and BLSE2018-86 are indicated (red and blue arrow respectively). Numbers indicate information of *fimH allele* (1), *bla*_CTX-M_ (2), VF clusters (3), geographic origin (4), source niche (5) and *presence/absence of cnf1* (6) for each strain. Lower inset (*) shows a close-up on EC131GY and BLSE2018-86 and surrounding strains.

**Sup. Figure 5: Characterization of EC131GY WT and mutant strain**

**A)** Representative Immunoblots anti-CNF1 and anti-HlyA showing levels of expression of both toxins in EC131GY WT and loss of CNF1 expression in EC131GY Δ*cnf1*::*kan^r^*  (left panel), as well as expression of CNF1-WT and CS in EC131GY Δ*cnf1* + pCNF1 WT and pCNF1-C866S (right panel). Immunoblots anti-RNA Polymerase (RNA Pol) show loading controls. **B)** Kinetics of individual growth monitored at OD_600_ of *E. coli* ST131 EC131GY WT (WT) and EC131GY Δ*cnf1*::*kan^r^*  (left graph) or EC131GY WT + pE, EC131GY Δ*cnf1* + pCNF1-WT and EC131GY Δ*cnf1* + pCNF1-CS. Data show one representative experiment performed with five biological replicates ± SD. **C)** Kinetic of growth competition *in vitro* between *E. coli* ST131 EC131GY WT and Δ*cnf1*::*kan^r^*  mixed 1:1. Bacteria were grown together for 5 hours at 37°C with shaking and CFU/ml determined on LB and LB kanamycin plates from serial dilutions. Each dot corresponds to the competitive index value (CI) between EC131GY WT and EC131GY Δ*cnf1*::*kan^r^*  (WT/ Δ*cnf1*::*kan^r^* ) at indicated time points. Data are shown as mean ± SEM, *n*=3 independent experiments. No significant difference, by Mann–Whitney U test.

**Sup. Figure 6: Immunomonitoring and bacterial invasion during UTI.**

**A-E)** Mice were infected separately with wild-type EC131GY (WT) or EC131GY Δ*cnf1*::*kan*^r^(Δ*cnf1::kan*^r^) via intravesical instillation of the bladder. Supernatants from homogenized infected bladders 24 hours post-infection were analysed using ELISA to determine cytokine levels in the tissue. The CFU from infected mice analysed here are shown in Figure 5. Graphs show levels of indicated cytokines **(A)**. Bladders were dissociated for flow cytometry 24 hours post-infection and immune cell populations were analyzed. Graphs show the percentage of each cell type indicated among all CD45+ immune cells present in the tissue **(B-E)**. No values were statistically significantly different between EC131GY (WT) or EC131GY Δ*cnf1*::*kan^r^*  (Δ*cnf1*::*kan*^r^) infection for cytokines or immune cell infiltration (Two-way ANOVA with Šidák’s multiple comparisons correction).

**Sup. Figure 7: Characterization of BLSE2018-86 WT and Δ*cnf1*::Kan^r^ mutant strain.**

**A)** Representative immunoblots anti-CNF1 and anti-HlyA showing levels of expression of the toxins in BLSE2018-86 WT and Δ*cnf1*::*kan*^r^. Immunoblots anti-RNA Polymerase (RNA Pol) show loading controls. **B)** Kinetics of individual growth monitored at OD_600_ of *E. coli* strains BLSE2018-86 WT and BLSE2018-86 Δ*cnf1*::*kan*^r^. Data show one representative experiment with 3 biological replicates ± SD. **C)** Kinetic of growth competition *in* *vitro* between *E. coli* strains BLSE2018-86 WT and BLSE2018-86 Δ*cnf1*::*kan*^r^ mixed 1:1. Bacteria were grown together and CFU/mL determined for each strain. Dots correspond to competitive index value (CI) between BSLE2018-86 WT and BLSE2018-86 Δ*cnf1*::*kan*^r^ at indicated time points. Data are shown as mean ± SD, *n*=3 independent experiments. No significant difference, by Mann–Whitney U test. **D)** For competition in the GIT, mice were concurrently infected via oral gavage with wildtype BLSE2018-86 (WT) and BLSE2018-86 Δ*cnf1*::*kan*^r^ (Δ*cnf1*::*kan*^r^). Levels of viable bacteria in feces were assessed at indicated times by measuring colony forming units (CFU), and competitive index value (CI) was calculated. Total of n=12, two replicates, ********P* < 0.001 by Wilcoxon signed-rank test.

**Sup. Table 1: *cnf1* and *hlyA* SNPs profile in ST131 genomes**

First two files correspond to the list of SNPs in *cnf1* (file1) and *hlyA* (file2) genes of ST131 genomes and distribution in profiles. The red square indicates the presence of a SNP in each profile. File 3 corresponds to the co-distribution of *cnf1* and *hlyA* SNPs profiles in ST131 genomes.

**Sup. Table 2: Profiles of acquired antibiotic-resistance genes and virulence factor encoding genes**

File 1 corresponds to the list of acquired antibiotic-resistance genes (RGs) and virulence factors (VFs) studied and their occurrence in the population of *E. coli* ST131 deposited in EnteroBase. Note that we retained RGs and VFs that show a differential occurrence in genomes, i.e. in less than 5,131 and more than 100 genomes. File 2 corresponds to the distribution of RGs and VFs profile (express as percentage) in strains within RG clusters and VF clusters.

**Sup. Table 3: Metadata describing *E. coli* genomes from EnteroBase**

**Sup. Table 4: List of representative sequences of CNF1 catalytic domain**

**Sup. Table 5: *E. coli* ST131 strains from the dataset and associated metadata**

**Sup. Table 6: List of strains and plasmids used in the study**

| Strains or plasmids | Characteristics | Source | Reference |
| --- | --- | --- | --- |
| EC131GY | Clinical isolate H1-001-0141-G-Y rendered Strep^R^ |  | ^1^ |
| EC131GY *Δcnf1::kan^r^* | Strep^R^, *cnf1* deletion via Kan^R^ insertion | This work |  |
| EC131GY*Δcnf1* | Strep^R^, *cnf1* deletion | This work |  |
| BLSE2018-86 | Clinical isolate rendered Strep^R^ |  | ^2^ |
| BLSE2018-86 *Δcnf1::kan^r^* | Strep^R^, *cnf1* deletion via Kan^R^ insertion | This work |  |
| pKD4 | Plasmid: Amp^R^; template for PCR of Kan^R^ | Coli Genetic Stock Center | ^3^ |
| pKOBEG | Plasmid: Cm^R^; Lambda Red recombinase system | Gift from JM. Ghigo | ^4^ |
| pCP20 | Plasmid: Flippase expression vector, Thermo sensible |  | ^5^ |
| pCNF1-WT | Plasmid: CNF1 gene under its own promoter cloned *BamH*I-*Kpn*I in p3xFlag-CmR | Kind gift from Petra Dersch | ^6^ |
| pCNF1-CS | Plasmid: C866S mutation introduced into pCNF1-WT | This work |  |
| pE | Plasmid: stuffer fragment inserted BamHI-KpnI in place of *cnf1* coding region in p3xFlag-CmR | This work |  |

**Sup. Table 7: List of primers**

| **Primers** | Sequences |
| --- | --- |
| cnf.H1-P1 | 5’_AGGTCTCTGTCTGAGAGTTATTCTCTGAATGCAGATGCCTCCGAAATATCGGTATTGAAGGTATTTTCAAAAAAATTTTGA |
| cnf.P2-H2 | 5’_TCAAAATTTTTTTGAAAATACCTTCAATACCGATATTTCGGAGGCATCTGCATTCAGAGAATAACTCTCAGACAGAGACCTGGTCCATATGAATATCCTCCTTAG |
| cnfver.fw | 5’_ATGGGTAACCAATGGCAACAAAAATATCTT |
| cnfver.rev | 5’_ATGGGTAACCAATGGCAACAAAAATATCTT |
| Q5C866Sfw | 5’_TCTAAGTGGTAGTACGACAATTG |
| Q5C866Srev | 5’_TTTCCGCTTGTAATGATTAC |
| VII009 | 5’_TCGTCTTCACCTCGAGGATCCTAATGGGGTTCAGATAATAC |
| VII054 | 5’_TAGTCACCACCTGCAGGTACCCaAAATTTTTTTGAAAATAC |

**Sup. Table 8: Scoary results**

**Sup. Table 9: Antibodies used for flow cytometry**

| **Molecule** | **Clone** | **Vendor** |
| --- | --- | --- |
| CD45 | 30-F11 | BD Biosciences |
| CD64 | X54-5/7.1.1 | BD Biosciences |
| CD103 | M290 | BD Biosciences |
| CD11b | M1/70 | BD Biosciences |
| CD11c | HL3 | BD Biosciences |
| F4/80 | CI:A3-1 | BIO-RAD |
| Ly6C and Ly6G (Gr1 antibody) | RB6-8C5 | BD Biosciences |
| MHC-II (I-A/I-E) | M5/114.15.2 | Invitrogen |
| SiglecF | E50-2440 | BD Biosciences |
| CD3 | 145-2C11 | BD Biosciences |
| CD4 | RM4-5 | BD Biosciences |
| IL-4Rα (CD124) | mIL4R-M1 | BD Biosciences |
| NK1.1 | PK136 | BD Biosciences |
| CD36 | HM36 | Biolegend |
| γδ TCR | GL3 | BD Biosciences |

**References**

1. de Lastours V, Laouénan C, Royer G et al. Mortality in *Escherichia coli* bloodstream infections: antibiotic resistance still does not make it. J Antimicrob Chemother. 2020;75:2334-2343.

2. Bonnet R, Beyrouthy R, Haenni M, Nicolas-Chanoine MH, Dalmasso G, Madec JY. Host Colonization as a Major Evolutionary Force Favoring the Diversity and the Emergence of the Worldwide Multidrug-Resistant *Escherichia coli* ST131. mBio. 2021;12:e0145121.

3. Datsenko KA, Wanner BL. One-step inactivation of chromosomal genes in *Escherichia coli* K-12 using PCR products. Proc Natl Acad Sci U S A. 2000;97:6640-6645.

4. Derbise A, Lesic B, Dacheux D, Ghigo JM, Carniel E. A rapid and simple method for inactivating chromosomal genes in Yersinia. FEMS Immunol Med Microbiol. 2003;38:113-116.

5. Cherepanov PP, Wackernagel W. Gene disruption in *Escherichia coli*: TcR and KmR cassettes with the option of Flp-catalyzed excision of the antibiotic-resistance determinant. Gene. 1995;158:9-14.

6. Chaoprasid P, Lukat P, Mühlen S et al. Crystal structure of bacterial cytotoxic necrotizing factor CNF_Y_ reveals molecular building blocks for intoxication. EMBO J. 2021;40:e105202.
