# Supplementary material for: The cnf1 gene is associated with an expanding Escherichia coli ST131 H30Rx/C2 subclade and confers a competitive advantage for gut colonization
Source: Gut Microbes. 2022 Sep 25;14(1):2121577. doi: 10.1080/19490976.2022.2121577 (PMC9519008; doi:10.1080/19490976.2022.2121577)
Supplement: Supplemental Material [file KGMI_A_2121577_SM6574.zip › 2121577_supplementary New/Tsoumtsa_et_al_Supplemental_03082022SP_v17082022.pdf]

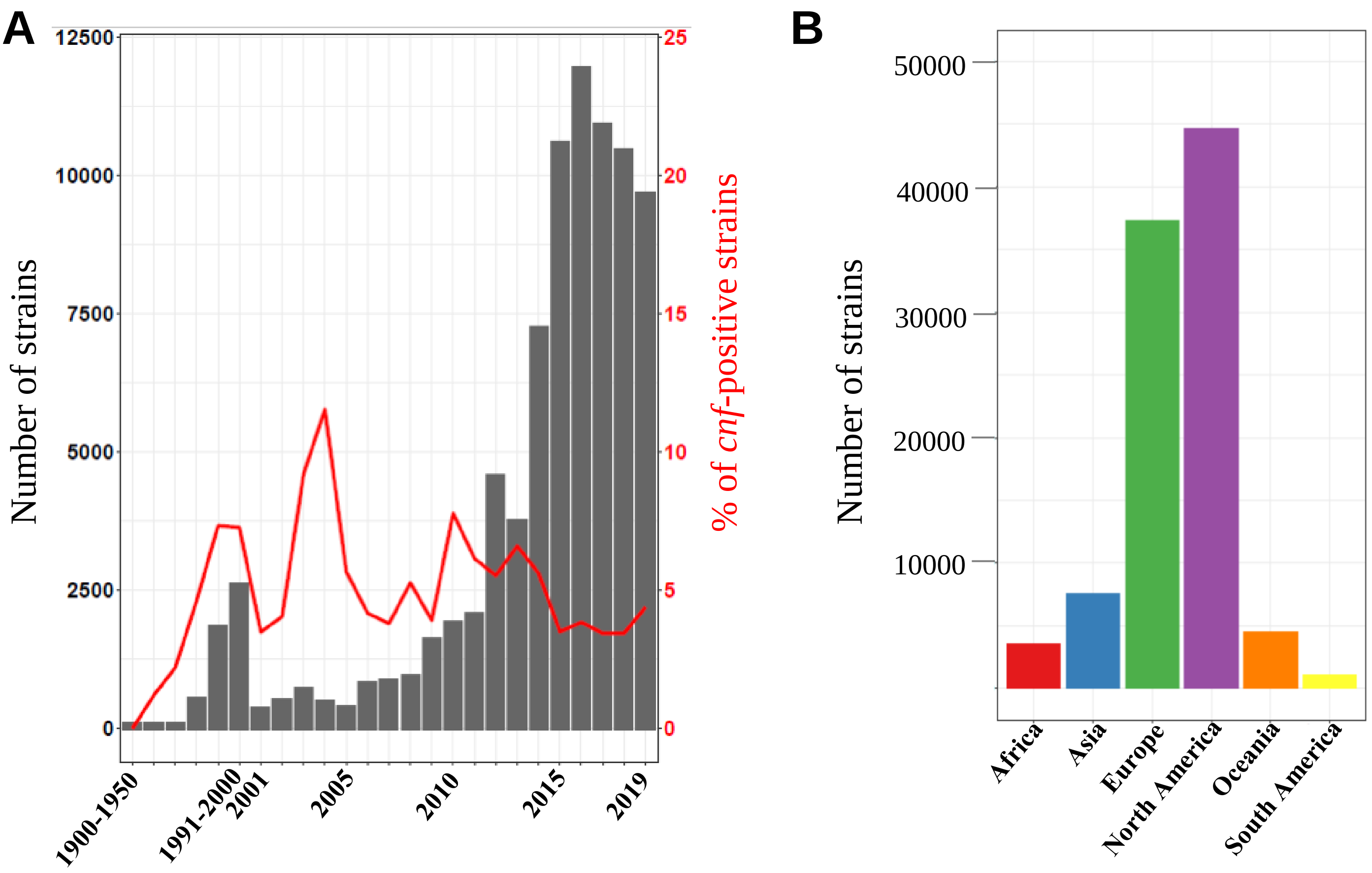

C

SOURCE NICHE	NEG	POS	TOTAL	*cnf1+* (%)
**Human**	45875	2643	48518	5,45
**Companion Animal**	1979	629	2608	24,12
**Animal Feed**	541	7	548	1,28
**Aquatic Animal**	400	9	409	2,20
**Environment**	6300	198	6498	3,05
**Food**	2227	58	2285	2,54
**Livestock**	13794	319	14113	2,26
**Poultry**	7919	59	7978	0,74
**Wild Animal**	2511	141	2652	5,32
**ND**	53099	2526	55625	4,54

D

PHYLOGROUPS	NEG	POS	TOTAL	*cnf1+* (%)
**A**	34982	0	34982	0,00
**B1**	37166	96	37262	0,26
**B2**	16891	5414	22305	24,27
**C**	3420	45	3465	1,30
**D**	9885	20	9905	0,20
**E**	16384	7	16391	0,04
**F**	2920	37	2957	1,25
**G**	1862	0	1862	0,00
**Clade I**	406	0	406	0,00
**Clade II**	6	0	6	0,00
**Clade III**	39	0	39	0,00
**Clade IV**	39	0	39	0,00
**Clade V**	166	0	166	0,00

Sup. Fig. 1 : Tsoumtsa et al.

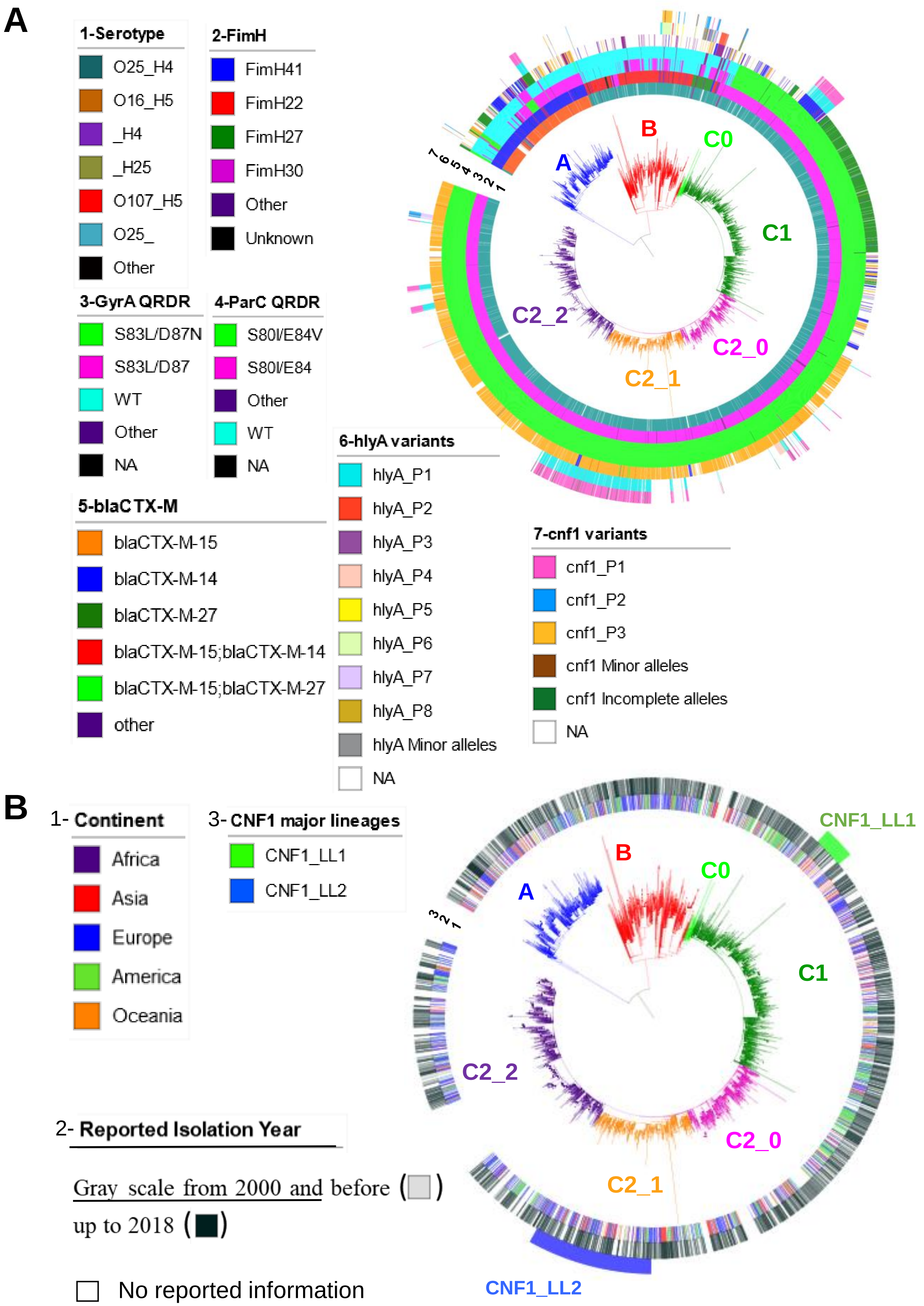

Sup. Fig. 2 : Tsoumtsa et al.

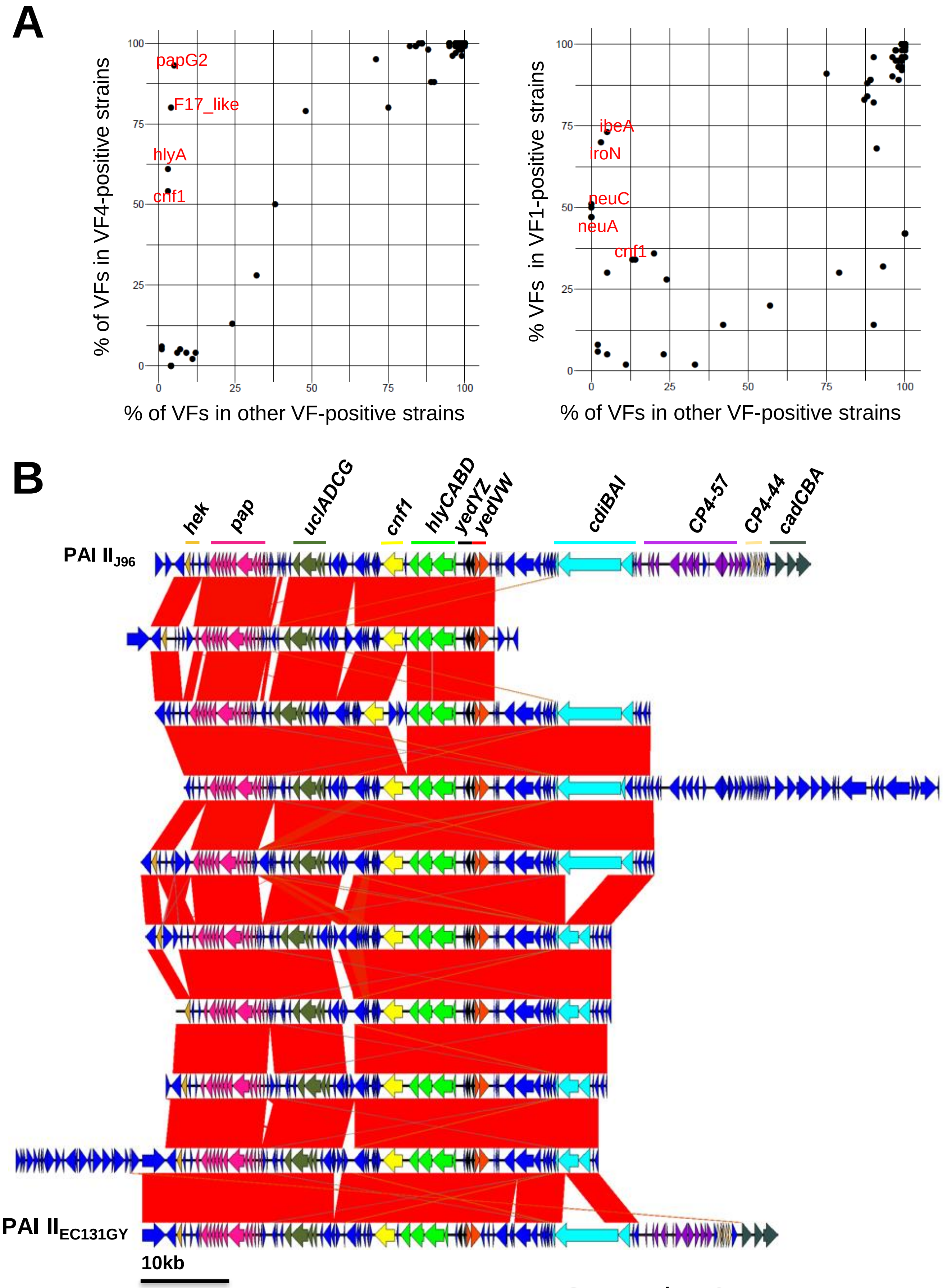

Sup. Fig. 3 : Tsoumtsa et al.

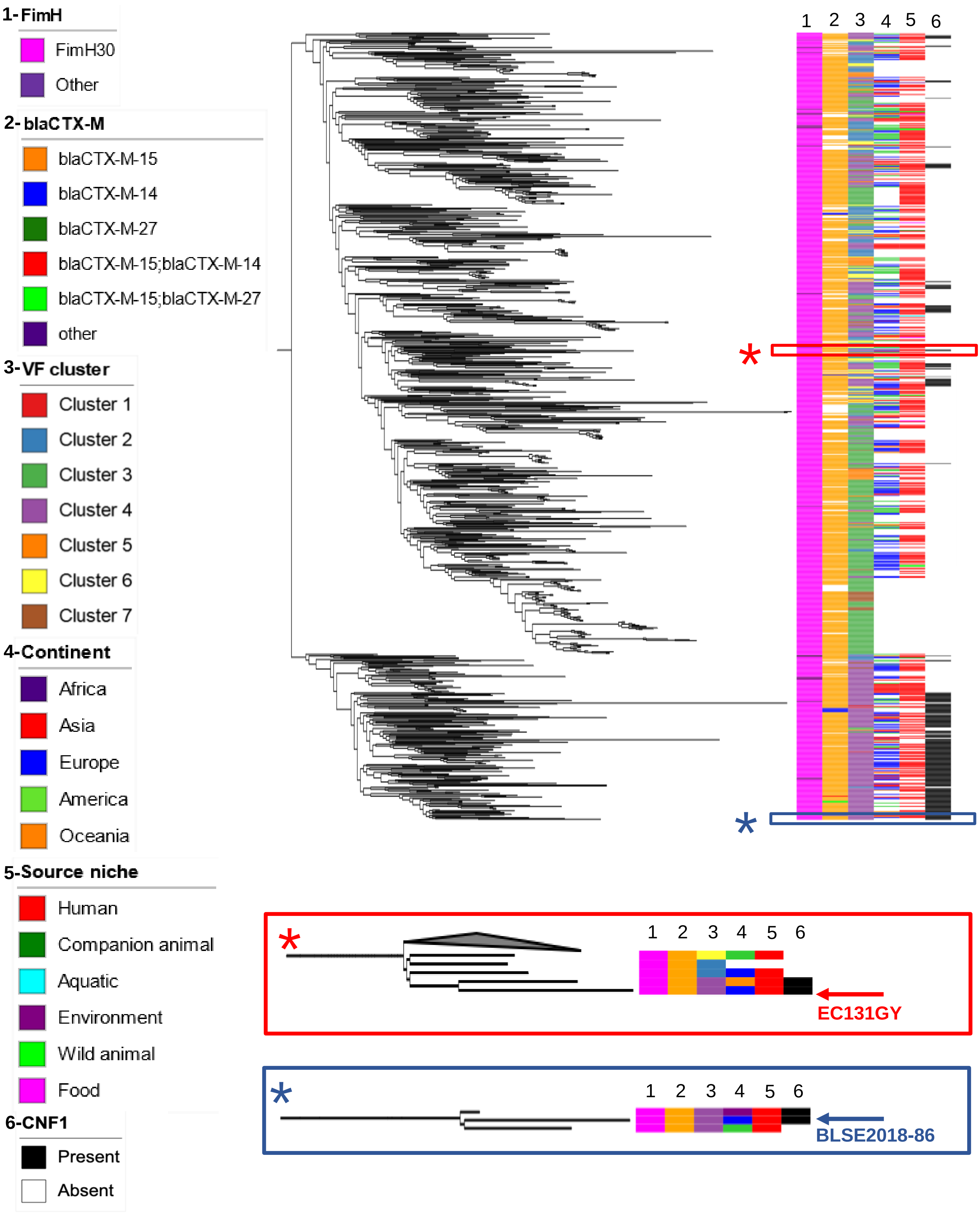

Sup. Fig. 4 : Tsoumtsa et al.

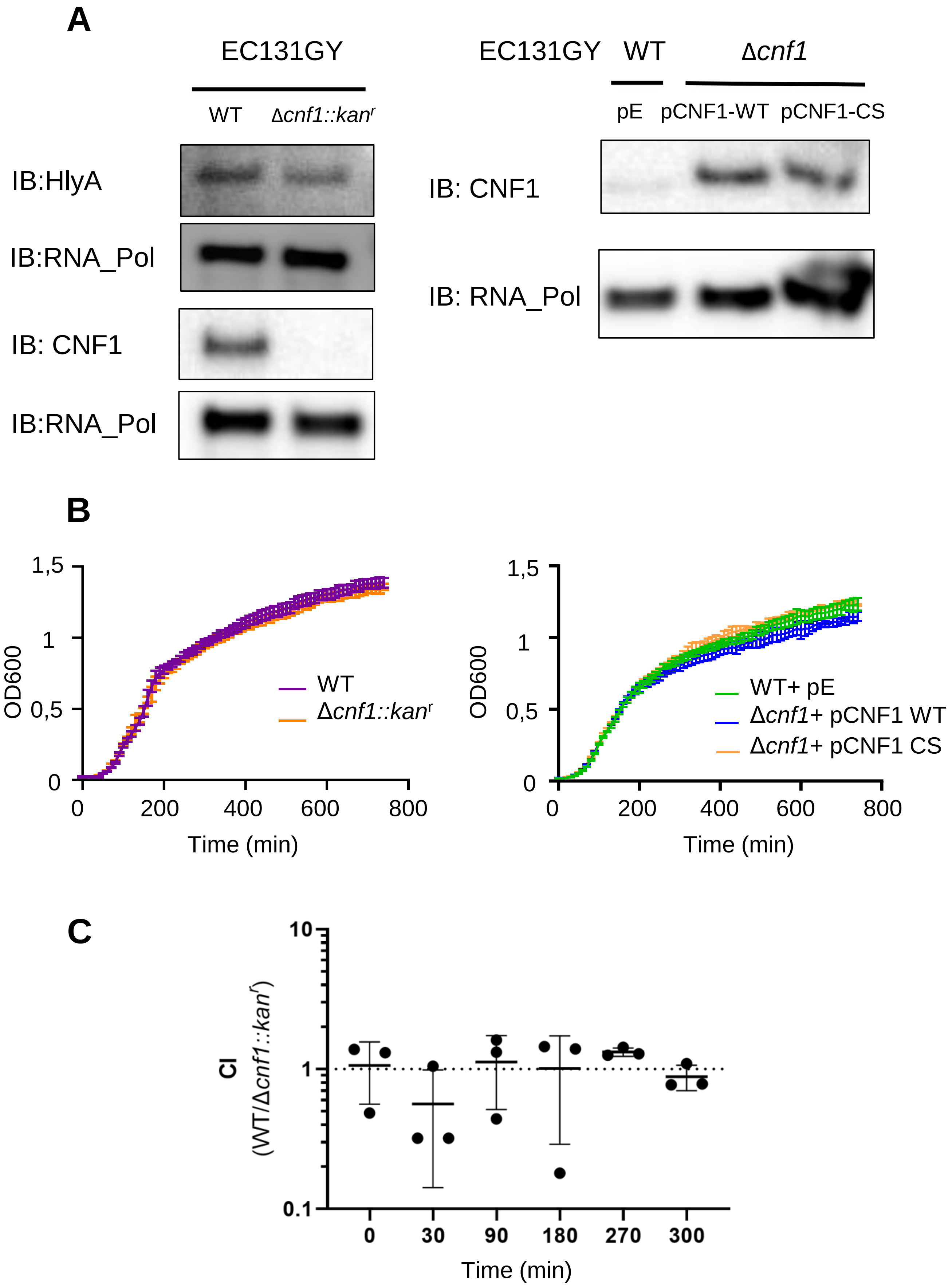

Sup. Fig. 5 : Tsoumtsa et al.

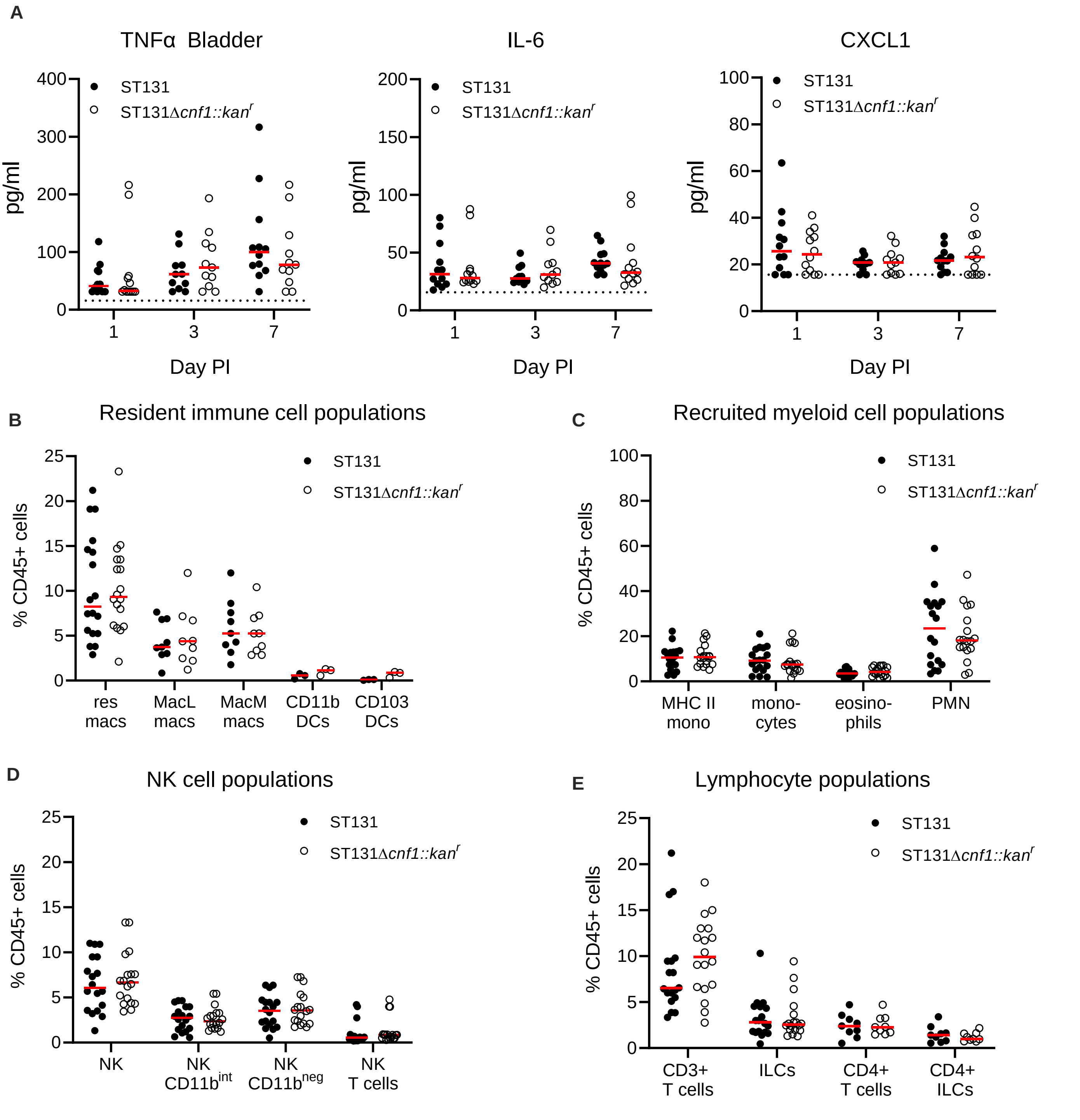

Sup. Fig. 6 : Tsoumtsa et al.

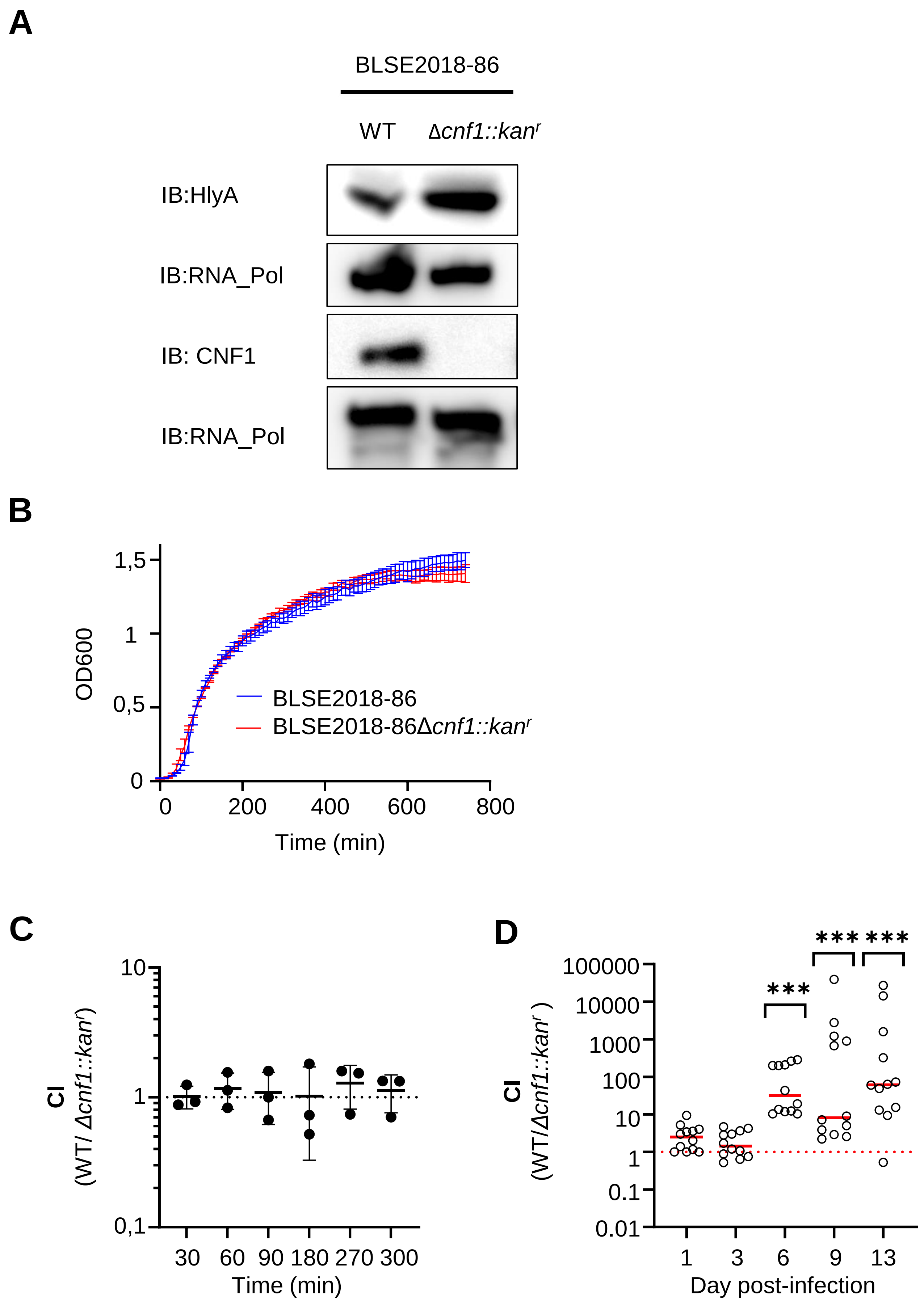

Sup. Fig. 7 : Tsoumtsa et al.
